# Assessing the performance of a serological point-of-care test in measuring detectable antibodies against SARS-CoV-2

**DOI:** 10.1371/journal.pone.0262897

**Published:** 2022-01-31

**Authors:** Peter V. Coyle, Reham Awni El Kahlout, Soha R. Dargham, Hiam Chemaitelly, Mohamed Ali Ben Hadj Kacem, Naema Hassan Abdulla Al-Mawlawi, Imtiaz Gilliani, Nourah Younes, Zaina Al Kanaani, Abdullatif Al Khal, Einas Al Kuwari, Andrew Jeremijenko, Anvar Hassan Kaleeckal, Ali Nizar Latif, Riyazuddin Mohammad Shaik, Hanan F. Abdul Rahim, Gheyath K. Nasrallah, Hadi M. Yassine, Mohamed G. Al Kuwari, Hamad Eid Al Romaihi, Patrick Tang, Roberto Bertollini, Mohamed H. Al-Thani, Laith J. Abu-Raddad

**Affiliations:** 1 Hamad Medical Corporation, Doha, Qatar; 2 Biomedical Research Center, Qatar University, Doha, Qatar; 3 Wellcome-Wolfson Institute for Experimental Medicine, Queens University, Belfast, United Kingdom; 4 Infectious Disease Epidemiology Group, Weill Cornell Medicine-Qatar, Cornell University, Qatar Foundation – Education City, Doha, Qatar; 5 World Health Organization Collaborating Centre for Disease Epidemiology Analytics on HIV/AIDS, Sexually Transmitted Infections, and Viral Hepatitis, Weill Cornell Medicine-Qatar, Cornell University, Qatar Foundation – Education City, Doha, Qatar; 6 College of Health Sciences, QU Health, Qatar University, Doha, Qatar; 7 Department of Biomedical Science, College of Health Sciences, Member of QU Health, Qatar University, Doha, Qatar; 8 Primary Health Care Corporation, Doha, Qatar; 9 Ministry of Public Health, Doha, Qatar; 10 Department of Pathology, Sidra Medicine, Doha, Qatar; 11 Department of Population Health Sciences, Weill Cornell Medicine, Cornell University, New York, New York, United States of America; University of Tripoli, LIBYA

## Abstract

This study investigated the performance of a rapid point-of-care antibody test, the BioMedomics COVID-19 IgM/IgG Rapid Test, in comparison with a high-quality, validated, laboratory-based platform, the Roche Elecsys Anti-SARS-CoV-2 assay. Serological testing was conducted on 709 individuals. Concordance metrics were estimated. Logistic regression was used to assess associations with seropositivity. SARS-CoV-2 seroprevalence was 63.5% (450/709; 95% CI 59.8%-67.0%) using the BioMedomics assay and 71.9% (510/709; 95% CI 68.5%-75.2%) using the Elecsys assay. There were 60 discordant results between the two assays, all of which were seropositive in the Elecsys assay, but seronegative in the BioMedomics assay. Overall, positive, and negative percent agreements between the two assays were 91.5% (95% CI 89.2%-93.5%), 88.2% (95% CI 85.1%-90.9%), and 100% (95% CI 98.2%-100%), respectively, with a Cohen’s kappa of 0.81 (95% CI 0.78–0.84). Excluding specimens with lower (Elecsys) antibody titers, the agreement improved with overall, positive, and negative percent concordance of 94.4% (95% CI 92.3%-96.1%), 91.8% (95% CI 88.8%-94.3%), and 100% (95% CI 98.2%-100%), respectively, and a Cohen’s kappa of 0.88 (95% CI 0.85–0.90). Logistic regression confirmed better agreement with higher antibody titers. The BioMedomics COVID-19 IgM/IgG Rapid Test demonstrated good performance in measuring detectable antibodies against SARS-CoV-2, supporting the utility of such rapid point-of-care serological testing to guide the public health responses and vaccine prioritization.

## Introduction

Coronavirus disease 2019 (COVID-19), caused by the novel severe acute respiratory syndrome coronavirus 2 (SARS-CoV-2), continues to present a global challenge, leading to health, social, and economic burdens [1]. Qatar experienced a large first SARS-CoV-2 epidemic wave in 2020, with a high rate of laboratory-confirmed infections at >60,000 infections per million population [2–4]. The wave predominantly affected the craft and manual workers who constitute just over half of Qatar’s total population [2]. Seroprevalence in this part of the population was measured at about 60% following this wave [5, 6].

Following this epidemic wave, Qatar’s public health authorities expanded serological testing for SARS-CoV-2 antibodies, for both healthcare and research purposes [6–8]. Moreover, antibody status was deliberated as one of the criteria for COVID-19 vaccine prioritization [9], and for a waiver of the quarantine requirement for international travelers [10].

To achieve more efficient, cost-effective, and widescale serological testing, the objective of this study was to compare the performance of a rapid point-of-care antibody test, the BioMedomics COVID-19 IgM/IgG Rapid Test [11], to a high-quality, validated, laboratory-based and automated assay, the Roche Elecsys Anti SARS-CoV-2 platform [12, 13], one of the most extensively used and investigated commercial platforms, having a specificity ≥99.8% [14, 15] and a sensitivity ≥89% [12, 14]. The relevance of this study is grounded on the utility of knowing antibody status as it can facilitate management of international travel [10], and importantly can optimize vaccination strategies, such as by delaying vaccination for those with prior infection [9], or by offering only one dose to those with a prior infection [16–18].

## Materials and methods

The study sample included 709 residual blood serum specimens that were collected and then tested for SARS-CoV-2 antibodies between October 10–21, 2020, from individuals receiving routine or other clinical care at Hamad Medical Corporation (HMC), the main provider of healthcare in Qatar, and the nationally designated provider for all COVID-19 healthcare needs. Qatar has a universal and modern healthcare system that is heavily subsidized and accessible to nationals and expatriate residents [8]. HMC provides the core of public healthcare services in Qatar, and has about 85% of the hospital bed capacity in the country. The 709 specimens used in this study were chosen from the residual blood serum specimens collected from outpatient and inpatient attendees at HMC [8].

Serological testing was performed using the Roche Elecsys Anti-SARS-CoV-2 (Roche, Switzerland) assay, a fully-automated electrochemiluminescent immunoassay [13], and the BioMedomics COVID-19 IgM/IgG Rapid Test (BioMedomics, Inc., United States of America), a lateral flow immunochromatographic assay [11].

The Roche Elecsys Anti-SARS-CoV-2 assay (hereafter “Elecsys”) uses a recombinant protein representing the nucleocapsid (N) antigen for determination of antibodies against SARS-CoV-2 [13]. Qualitative anti-SARS-CoV-2 results were generated following the manufacturer’s instructions (reactive for optical density (proxy for antibody titer [14]) cutoff index ≥1.0 vs. non-reactive for cutoff index <1.0) [13].

The BioMedomics COVID-19 IgM/IgG Rapid Test (hereafter “BioMedomics”) is a lateral flow immunoassay that contains a colloidal, gold-labeled, recombinant coronavirus antigen and a quality control antibody colloidal gold marker, two detection lines (IgG and IgM lines), and one quality control line (C) fixed on a nitrocellulose membrane [11]. The antigen used in this assay is SARS-CoV-2 MK201027 antigen that is found in the receptor binding domain of the spike protein [19]. Qualitative anti-SARS-CoV-2 results were generated by reading the detection line(s) [11].

Results of the serological testing were subsequently linked to the national centralized SARS-CoV-2 real-time reverse-transcription polymerase chain reaction (RT-PCR) testing and hospitalization database that includes records for all RT-PCR testing and COVID-19 hospitalizations in Qatar since the start of the epidemic [2]. The database also includes the severity classification of hospitalized cases based on individual chart reviews completed by trained medical personnel using the World Health Organization (WHO) criteria [20].

For the RT-PCR testing in Qatar [2, 5, 6, 8, 21, 22], nasopharyngeal and/or oropharyngeal swabs (Huachenyang Technology, China) are collected and placed in Universal Transport Medium (UTM). Aliquots of UTM are: extracted on a QIAsymphony platform (QIAGEN, USA) and tested with RT-qPCR using TaqPath^™^ COVID-19 Combo Kits (100% sensitivity and specificity [23]; Thermo Fisher Scientific, USA) on an ABI 7500 FAST (ThermoFisher, USA); extracted using a custom protocol [24] on a Hamilton Microlab STAR (Hamilton, USA) and tested using AccuPower SARS-CoV-2 Real-Time RT-PCR Kits (100% sensitivity and specificity [25]; Bioneer, Korea) on an ABI 7500 FAST; or loaded directly into a Roche cobas^®^ 6800 system and assayed with a cobas^®^ SARS-CoV-2 Test (95% sensitivity, 100% specificity [26]; Roche, Switzerland). The first assay targets the viral S, N, and ORF1ab regions. The second targets the viral RdRp and E-gene regions, and the third targets the ORF1ab and E-gene regions.

All RT-PCR tests were conducted at the HMC Central Laboratory or Sidra Medicine Laboratory, following standardized protocols.

Cross-tabulations of the serological testing results were conducted using the Elecsys assay as the reference standard. Concordance metrics were estimated and included the positive, negative, and overall percent agreements, noting that this study was not designed to assess sensitivity and specificity of the BioMedomics assay. In addition, Cohen’s kappa statistic [27] was estimated to measure the level of agreement, beyond chance, between the two diagnostic approaches. Ranging between 0 and 1, a kappa statistic <0.40 indicates poor agreement, a value between 0.40 and 0.75 denotes fair/good agreement, and a value >0.75 signifies excellent agreement [27]. Statistical significance was set at 5% and a 95% confidence interval (CI) was estimated for each metric.

Univariable and multivariable logistic regressions were conducted to assess associations between seropositivity using the BioMedomics assay and each of the following covariates: RT-PCR cycle threshold (Ct) value, optical density value of the Elecsys assay result, and severity of infection. Covariates with p-values ≤0.2 in the univariable regression analysis were included in the multivariable model. Covariates with p-values ≤0.05 in the multivariable analysis were regarded as covariates with strong evidence for an association with the outcome. Analyses were performed using Microsoft Excel and IBM-SPSS version 26.0.

The research methods were approved on June 3, 2020 by the ethics review boards at HMC (HMC IRB number MRC-05-133) and Weill Cornell Medicine-Qatar (WCM-Q IRB number 21–00001), with waiver of informed consent.

## Results

Of the 709 individuals included in this study and were tested for SARS-CoV-2 antibodies, the majority were men (63.0%) and of Indian (29.5%) and Nepalese (14.1%) nationalities (Table 1). Most (81.9%) were 20–49 years of age, with the median age being 36 years (interquartile range [IQR]: 30–45) (Table 1).

**Table 1:** Demographic characteristics of the sample that included 709 individuals who were tested for SARS-CoV-2 antibodies using the BioMedomics COVID-19 IgM/IgG Rapid Test and the Roche Elecsys Anti-SARS-CoV-2 assay.

	N (%)
**Age**	
<20 years	14 (2.0)
20–29 years	143 (20.2)
30–39 years	276 (38.9)
40–49 years	162 (22.8)
50–59 years	81 (11.4)
60–69 years	22 (3.1)
≥70 years	11 (1.6)
**Sex**	
Female	262 (37.0)
Male	447 (63.0)
**Nationality**	
Bangladeshi	53 (7.5)
Filipino	84 (11.8)
Indian	209 (29.5)
Nepalese	100 (14.1)
Qatari	55 (7.8)
Other nationalities	208 (29.3)

Seroprevalence of SARS-CoV-2-IgG in this sample was estimated at 63.5% (450/709; 95% CI 59.8%-67.0%) using the BioMedomics assay and at 71.9% (510/709; 95% CI 68.5%-75.2%) using the Elecsys assay. Seroprevalence of SARS-CoV-2-IgM was estimated at 8.5% (60/709; 95% CI 6.5%-10.8%), measured only using the BioMedomics assay. Results of serological testing for each of the 709 individuals are tabulated in S1 Table.

**Fig 1:**
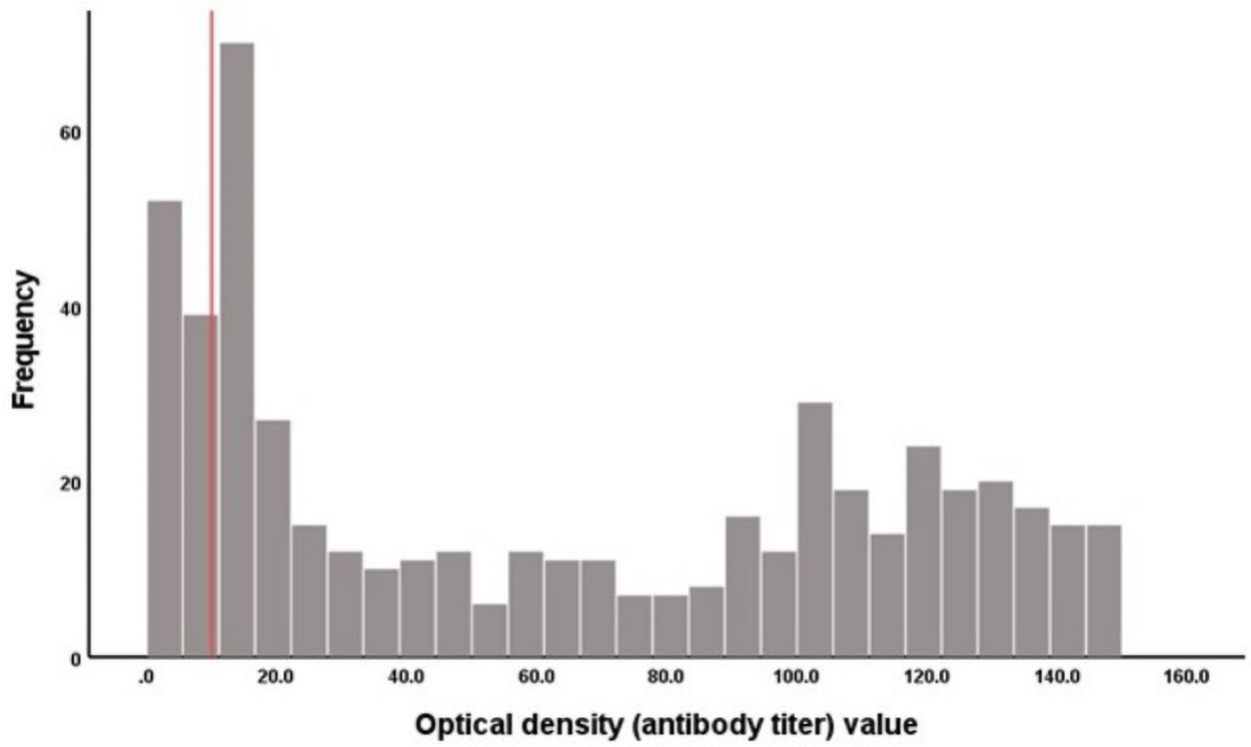
Distribution of optical density (antibody titer) values among 510 specimens that were seropositive in the Roche Elecsys Anti-SARS-CoV-2 test. The red line represents the threshold value of ‘10’ that stratifies the distribution of Elecsys optical density values into lower and higher antibody titer categories.

Among those seropositive in the Elecsys assay, optical density values (antibody titers) ranged between 1.0 and >150.0 with a median of 55.7 (Fig 1). There were 60 discordant results between the two assays; all of which were seropositive in the Elecsys assay, but seronegative in the BioMedomics assay (8.5%). None of the specimens was seropositive in the BioMedomics assay, but seronegative in the Elecsys assay. One person (patient 509) was (borderline) seropositive in the Elecsys assay before being diagnosed as RT-PCR positive (S1 Table), possibly due to prolonged RT-PCR positivity [28].

The percentage of individuals who had an RT-PCR-confirmed SARS-CoV-2 diagnosis *prior* to the serological testing was 37.1% (263/709; 95% CI 33.5%-40.8%) (S1 Table). The median difference in days between the prior RT-PCR diagnosis and the serological test was 123 days (IQR: 66–156). The RT-PCR Ct values ranged between 12.5 and 38.3 with a median of 21.9. Among those seropositive in the BioMedomics assay, 53.3% (240/450; 95% CI 48.6%-58.0%) had received a prior RT-PCR-positive result. Ct values ranged between 12.5 and 38.3 with a median of 21.8. Among those seropositive in the Elecsys assay, 51.0% (260/510; 95% CI 46.6%-55.4%) had received a prior RT-PCR-positive result. Ct values ranged between 12.5 and 38.3 with a median of 22.0. Among the 60 discordant specimens, 33.3% (20/60; 95% CI 21.7%-46.7%) had a prior RT-PCR-positive result. Ct values ranged between 13.7 and 36.8 with a median of 23.1.

Among those with a prior RT-PCR-positive result *and* seropositive in the Elecsys assay, 92.3% (241/261; 95% CI 88.4%-95.3%) were seropositive in the BioMedomics assay. Two individuals were seronegative in both antibody assays, but had a prior RT-PCR-confirmed SARS-CoV-2 diagnosis (S1 Table). The first individual was diagnosed on May 21, 2020 (Ct value was 35.7), 153 days prior to the blood collection date. With the high Ct value, one cannot exclude the possibility of an RT-PCR false positive result. The second individual was diagnosed on October 13, 2020 (Ct value was 15.6), only four days prior to the blood collection date, and thus the blood was probably drawn too early to detect development of antibodies.

Data on COVID-19 disease severity per the WHO classification (derived from the national COVID-19 hospitalization database [2, 29]) were available for 47 persons: 7 individuals were classified as mild, 19 were classified as moderate, 14 were classified as severe, and 7 were classified as critical (S1 Table). For all other individuals no severity classification was conducted, due to absence of serious symptoms to require hospitalization and severity assessment, and thus the infection can be assumed to be asymptomatic or mild. No COVID-19 deaths were reported among study participants.

The overall, positive, and negative percent agreements between the two assays were estimated at 91.5% (95% CI 89.2%-93.5%), 88.2% (95% CI 85.1%-90.9%), and 100% (95% CI 98.2%-100%), respectively (Table 2). Cohen’s kappa statistic was estimated at 0.81 (95% CI 0.78–0.84) indicating “excellent” agreement [27] between the two assays (Table 2).

**Table 2:** Concordance metrics between two SARS-CoV-2 antibody assays: The BioMedomics COVID-19 IgM/IgG Rapid Test and the Roche Elecsys Anti-SARS-CoV-2 including A) all negative and positive specimens, B) negative specimens on Elecsys and specimens with higher Elecsys antibody titers (excluding specimens with Elecsys optical density values <10), and C) negative specimens on Elecsys and specimens with lower Elecsys antibody titers (excluding specimens with Elecsys optical density values ≥10).

A)		**Roche Elecsys Anti-SARS-CoV-2**			**Overall percent agreement**	**Positive percent agreement**	**Negative percent agreement**	**Cohen’s kappa statistic**
		**Positive**	**Negative**	**Total**	**% (95% CI)**	**% (95% CI)**	**% (95% CI)**	**k (95% CI)**
**BioMedomics COVID-19 IgM/IgG Rapid Test**	**Positive**	450	0	450	91.5% (89.2%-93.5%)	88.2% (85.1%-90.9%)	100% (98.2%-100%)	0.81 (0.78–0.84)
	**Negative**	60	199	259				
	**Total**	510	199	709				
B)		**Roche Elecsys Anti-SARS-CoV-2**			**Overall percent agreement**	**Positive percent agreement**	**Negative percent agreement**	**Cohen’s kappa statistic**
		**Positive**	**Negative**	**Total**	**% (95% CI)**	**% (95% CI)**	**% (95% CI)**	**k (95% CI)**
**BioMedomics COVID-19 IgM/IgG Rapid Test**	**Positive**	394	0	394	94.4% (92.3%-96.1%)	91.8% (88.8%-94.3%)	100% (98.2%-100%)	0.88 (0.85–0.90)
	**Negative**	35	199	234				
	**Total**	429	199	628				
C)		**Roche Elecsys Anti-SARS-CoV-2**			**Overall percent agreement**	**Positive percent agreement**	**Negative percent agreement**	**Cohen’s kappa statistic**
		**Positive**	**Negative**	**Total**	**% (95% CI)**	**% (95% CI)**	**% (95% CI)**	**k (95% CI)**
**BioMedomics COVID-19 IgM/IgG Rapid Test**	**Positive**	56	0	56	91.1% (87.1%-94.1%)	69.1% (57.9%-78.9%)	100% (98.2%-100%)	0.76 (0.70–0.82)
	**Negative**	25	199	224				
	**Total**	81	199	280				

Including only specimens taken ≥14 days after the prior RT-PCR-confirmed infection, the overall, positive, and negative percent agreements between the two assays were estimated at 91.8% (95% CI 89.5%-93.8%), 88.5% (95% CI 85.3%-91.2%), and 100% (95% CI 98.1%-100%), respectively (S2 Table). Cohen’s kappa statistic was estimated at 0.82 (95% CI 0.78–0.85) indicating “excellent” agreement [27] between the two assays (S2 Table).

Including only specimens with higher antibody titers in the comparison (i.e., excluding specimens with low Elecsys optical density values <10), the Cohen’s kappa statistic was estimated at 0.88 (95% CI 0.85–0.90) indicating “excellent” agreement [27] between the two assays, and at higher value compared to the result for the full sample (Table 2). Including only specimens with low antibody titers in the comparison (i.e. excluding specimens with Elecsys optical density values ≥10), the Cohen’s kappa statistic was estimated at 0.76 (95% CI 0.70–0.82) also indicating “excellent” agreement [27] between the two assays, but still an inferior agreement compared to the result for the subsample of higher antibody titers, and for the full sample (Table 2). The cutoff at Elecsys optical density value of 10 to distinguish low from higher antibody titers was informed by the distribution of the optical density values (Fig 1). Specimens with Elecsys optical density value <10 constituted 15.9% (81/510) of all seropositive specimens by the Elecsys assay.

The multivariable logistic regression identified significant associations between seropositivity using the BioMedomics assay and both the Elecsys optical density value and the RT-PCR Ct value or its absence (that is no prior RT-PCR-confirmed infection), but no association with the severity of the infection (Table 3). The adjusted odds ratio (aOR) of seropositivity was 6.14 (95% CI 3.31–11.4, p<0.001) for those with higher antibody titers (Elecsys optical density values ≥10) compared to those with low antibody titers (Elecsys optical density values <10), reflecting much higher agreement between the two measures for specimens with higher antibody titers.

**Table 3:** Results of univariable and multivariable logistic regression, assessing the association between seropositivity using the BioMedomics COVID-19 IgM/IgG Rapid Test and the following covariates: RT-PCR Ct value (cut-off at 30), Elecsys optical density value, and severity of infection.

		Crude OR (95% CI)	P-value	aOR (95% CI)	P-value
**RT-PCR cycle threshold (Ct) value**	**Low Ct value (<30)**	Ref		Ref	
	**High Ct value (≥30)**	0.32 (0.13–0.79)	0.013	0.24 (0.09–0.65)	0.005
	**No RT-PCR/No Ct value**	0.07 (0.04–0.13)	<0.001	0.27 (0.13–0.56)	<0.001
**Optical density value of the Roche Elecsys Anti SARS-CoV-2 assay**	**Lower antibody titers (optical density value <10.0)**	Ref		Ref	
	**Higher antibody titers (≥10.0)**	5.03 (2.80–9.02)	<0.001	6.14 (3.31–11.4)	<0.001
	**Negative result (no optical density value)**	N/A	N/A	N/A	N/A
**Severity** ^$^	**Non-severe (asymptomatic, mild, or moderate infection)**	Ref		Ref	
	**Severe (severe or critical infection)**	5.68 (1.31–24.58)	0.020	1.11 (0.22–5.58)	0.899

OR-odds ratio; aOR-adjusted odds ratio; CI-confidence interval.

^$^Severity per WHO classification [20].

For individuals where no severity classification was conducted, due to absence of serious symptoms requiring hospitalization and severity assessment, infection was assumed to be asymptomatic or mild.

The aOR of seropositivity was 0.24 (95% CI 0.09–0.65, p = 0.005) for those with a Ct value ≥30 compared to those with a Ct value <30, possibly because of some RT-PCR false positivity measures for those with higher Ct values. The aOR of seropositivity was 0.27 (95% CI 0.13–0.56, p<0.001) for those with no Ct value (that is no prior RT-PCR-confirmed infection), reflecting the lower likelihood of having been exposed to the infection if they were never diagnosed with the infection using RT-PCR.

A sensitivity analysis for the multivariable logistic regression, using the Ct value cut-off of 35 instead of 30, confirmed the same results and suggested higher occurrence of RT-PCR false positive results when the Ct value is ≥35 than when the Ct value is ≥30 (S3 Table).

## Discussion

The above results document good performance of the BioMedomics rapid test in measuring detectable antibodies against SARS-CoV-2. The performance of this point-of-care test was also as expected for a rapid test: it was optimal when antibody titers were high, but less optimal when they were low. However, this should not be a hindrance for its use in facilitating optimal vaccination strategies, such as delaying vaccination for those with a record of prior infection [9], or offering only one vaccine dose to those who are seropositive [16–18]. Given the good performance of the BioMedomics assay, this assay can be used for a rapid assessment of prior exposure whether in clinical settings or for self-testing, as also seen in other published studies for this assay [19, 25].

These findings demonstrate the utility of this assay to assess past SARS-CoV-2 infection and seropositivity as a marker of immunity against infection, particularly considering the growing evidence indicating that natural infection elicits strong protection against reinfection that lasts for at least several months, even against variants of concern [10, 21, 22, 30–32]. These findings also support the concept of using rapid antibody testing for more efficient, cost-effective, and widescale serological testing to guide vaccine prioritization, or possible issuance of immunity passports, given supporting evidence for protection against infection (and not only disease) after prior infection or vaccination [10, 33–35].

This study has several limitations. We investigated the performance of serological testing on stored serum specimens and not on whole blood, and thus the real-world performance of this rapid assay in a point-of-care setting might be lower. The performance of the rapid assay was compared to a laboratory-based assay that uses a different antigen target, the Roche Elecsys Anti SARS-CoV-2 platform (and to RT-PCR testing), but not to a gold standard neutralization test, as such test was not available to the study investigators. Having said so, the Elecsys assay is one of the most extensively used and investigated commercial platforms, having a specificity ≥99.8% [13–15] and a sensitivity ≥89% [12, 14]. Therefore, it is not likely that this limitation could have affected the findings.

False negative results could have been due to waning of antibody titers over time, especially with a long delay between the primary infection and the antibody test. Although Cohen’s kappa is a conventional measure of agreement between assays, its performance can vary with the variation in infection prevalence in the population, despite equal test performance in terms of sensitivity and specificity. Despite the high kappa values qualifying the agreement between the two assays as “excellent”, the BioMedomics assay missed detection of SARS-CoV-2 antibodies in >10% of seropositive specimens by Elecsys.

We did not investigate the effects of subjective elements on the results’ interpretation, such as by different individuals implementing, reading, and interpreting each test separately on the same blood specimen. Nonetheless, there does not appear to be strong reasons to suspect interference of subjective elements on the results’ interpretation. Effect of disease severity on antibody titers was not investigated as the number of severe cases was low in the study sample—the epidemic in Qatar had relatively low severity with the young and working-age demographic structure of the population [2, 4, 36]. Test performance by time since infection was not investigated as record of prior infection was available only for a subset of seropositive individuals. Test performance was investigated for natural-infection antibodies, but not for vaccine-induced antibodies. Such lines of further investigation might be addressed in future studies.

In conclusion, the BioMedomics rapid point-of-care test demonstrated good performance in measuring detectable antibodies against SARS-CoV-2, with better performance for specimens with higher antibody titers, demonstrating the utility of such assays in mass expansion of serological testing to guide public health responses and vaccine prioritization.

## Supporting information

S1 Table Characteristics of all specimens tested using the BioMedomics COVID-19 IgM/IgG Rapid Test and the Roche Elecsys Anti SARS-CoV-2 assay. (DOCX)

S2 Table Concordance metrics between two SARS-CoV-2 antibody assays: The BioMedomics COVID-19 IgM/IgG Rapid Test and the Roche Elecsys Anti-SARS-CoV-2 including only specimens taken ≥14 days after the prior RT-PCR-confirmed infection. (DOCX)

S3 Table Results of univariable and multivariable logistic regression, assessing the association between seropositivity using the BioMedomics COVID-19 IgM/IgG Rapid Test and the following covariates: RT-PCR Ct value (cut-off at 35), Elecsys optical density value, and severity of infection. (DOCX)
