# Supplementary material for: Assessing the performance of a serological point-of-care test in measuring detectable antibodies against SARS-CoV-2
Source: PLoS One. 2022 Jan 31;17(1):e0262897. doi: 10.1371/journal.pone.0262897 (PMC8803198; doi:10.1371/journal.pone.0262897)
Supplement: S1 Table — (DOCX) [file pone.0262897.s001.docx]

**Assessing the performance of a serological point-of-care test in measuring detectable antibodies against SARS-CoV-2**

Supporting Information

**Table S1.** Characteristics of all specimens tested using the BioMedomics COVID-19 IgM/IgG Rapid Test and the Roche Elecsys Anti SARS-CoV-2 assay.

| **Specimen Number^#^** | **Date of blood collection for antibody testing** | **BioMedomics COVID-19 IgM/IgG Rapid Test^*^** | | **Roche Elecsys Anti SARS-CoV-2^**^** | | **PCR testing prior to the serological test** | | **Severity^$^** |
| --- | --- | --- | --- | --- | --- | --- | --- | --- |
|  |  | **IgM Test result** | **IgG Test result** | **Optical density value (antibody titers)** | **Test result** | **Date of first**  **PCR-positive test (if any)** | **Ct value** |  |
| 1 | 15-Oct-20 | Negative | Positive | >150 | Positive |  |  |  |
| 2 | 11-Oct-20 | Negative | Positive | >150 | Positive |  |  |  |
| 3 | 15-Oct-20 | Negative | Positive | >150 | Positive |  |  |  |
| 4 | 18-Oct-20 | Negative | Positive | >150 | Positive |  |  |  |
| 5 | 18-Oct-20 | Positive | Positive | >150 | Positive |  |  |  |
| 6 | 13-Oct-20 | Negative | Positive | >150 | Positive | 1-May-20 | 19.23 | Severe |
| 7 | 12-Oct-20 | Negative | Positive | >150 | Positive | 16-Apr-20 | 19.07 |  |
| 8 | 12-Oct-20 | Negative | Positive | >150 | Positive | 4-Apr-20 |  |  |
| 9 | 18-Oct-20 | Negative | Positive | 148 | Positive |  |  |  |
| 10 | 18-Oct-20 | Negative | Positive | 148 | Positive |  |  |  |
| 11 | 13-Oct-20 | Negative | Positive | 148 | Positive | 30-May-20 | 17.38 |  |
| 12 | 18-Oct-20 | Negative | Positive | 147 | Positive |  |  |  |
| 13 | 19-Oct-20 | Negative | Positive | 146 | Positive |  |  |  |
| 14 | 18-Oct-20 | Positive | Positive | 146 | Positive |  |  |  |
| 15 | 18-Oct-20 | Negative | Positive | 145 | Positive |  |  |  |
| 16 | 19-Oct-20 | Negative | Positive | 144 | Positive | 16-Jun-20 | 33.04 | Moderate |
| 17 | 15-Oct-20 | Positive | Positive | 144 | Positive | 20-May-20 | 26.38 | Moderate |
| 18 | 18-Oct-20 | Negative | Positive | 143 | Positive |  |  |  |
| 19 | 19-Oct-20 | Negative | Positive | 143 | Positive | 2-Jul-20 | 18.56 |  |
| 20 | 19-Oct-20 | Negative | Positive | 142 | Positive |  |  |  |
| 21 | 19-Oct-20 | Negative | Positive | 142 | Positive | 21-Jul-20 | 34.77 |  |
| 22 | 18-Oct-20 | Positive | Positive | 141 | Positive | 13-Jul-20 | 15.32 | Moderate |
| 23 | 18-Oct-20 | Negative | Positive | 141 | Positive | 17-Jun-20 | 33.52 |  |
| 24 | 18-Oct-20 | Negative | Positive | 141 | Positive | 27-May-20 | 22.8 |  |
| 25 | 16-Oct-20 | Negative | Negative | 140 | Positive |  |  |  |
| 26 | 19-Oct-20 | Negative | Positive | 140 | Positive | 7-Jun-20 | 20.16 |  |
| 27 | 18-Oct-20 | Negative | Positive | 140 | Positive | 1-Jun-20 | 16.05 |  |
| 28 | 18-Oct-20 | Negative | Positive | 140 | Positive | 25-Apr-20 | 15.46 |  |
| 29 | 18-Oct-20 | Negative | Positive | 139 | Positive | 8-Jul-20 | 18 |  |
| 30 | 18-Oct-20 | Negative | Positive | 139 | Positive | 27-Jun-20 | 24.77 |  |
| 31 | 19-Oct-20 | Negative | Positive | 138 | Positive |  |  |  |
| 32 | 18-Oct-20 | Positive | Positive | 138 | Positive |  |  |  |
| 33 | 19-Oct-20 | Negative | Positive | 138 | Positive | 8-Aug-20 | 24.55 |  |
| 34 | 18-Oct-20 | Negative | Positive | 137 | Positive |  |  |  |
| 35 | 18-Oct-20 | Negative | Positive | 137 | Positive |  |  |  |
| 36 | 18-Oct-20 | Negative | Positive | 137 | Positive |  |  |  |
| 37 | 19-Oct-20 | Negative | Positive | 137 | Positive |  |  |  |
| 38 | 19-Oct-20 | Negative | Positive | 137 | Positive |  |  |  |
| 39 | 19-Oct-20 | Positive | Positive | 137 | Positive |  |  |  |
| 40 | 18-Oct-20 | Negative | Positive | 137 | Positive | 12-Jul-20 | 17.8 |  |
| 41 | 14-Oct-20 | Positive | Positive | 136 | Positive | 20-May-20 | 18.78 |  |
| 42 | 18-Oct-20 | Negative | Positive | 135 | Positive |  |  |  |
| 43 | 18-Oct-20 | Negative | Positive | 135 | Positive |  |  |  |
| 44 | 18-Oct-20 | Positive | Positive | 135 | Positive | 2-Jul-20 | 19.99 | Moderate |
| 45 | 18-Oct-20 | Negative | Positive | 134 | Positive |  |  |  |
| 46 | 16-Oct-20 | Positive | Positive | 134 | Positive |  |  |  |
| 47 | 18-Oct-20 | Negative | Positive | 134 | Positive | 6-Aug-20 | 26.9 |  |
| 48 | 19-Oct-20 | Negative | Positive | 133 | Positive |  |  |  |
| 49 | 19-Oct-20 | Negative | Positive | 133 | Positive | 29-Jul-20 | 16.54 |  |
| 50 | 19-Oct-20 | Negative | Positive | 133 | Positive | 28-Jul-20 | 30.99 |  |
| 51 | 18-Oct-20 | Negative | Positive | 132 | Positive |  |  |  |
| 52 | 12-Oct-20 | Negative | Positive | 132 | Positive |  |  |  |
| 53 | 14-Oct-20 | Negative | Positive | 132 | Positive | 20-Jun-20 | 20.66 |  |
| 54 | 18-Oct-20 | Negative | Positive | 132 | Positive | 19-Apr-20 | 20.88 | Moderate |
| 55 | 18-Oct-20 | Negative | Positive | 131 | Positive | 10-Jun-20 | 30.06 |  |
| 56 | 13-Oct-20 | Negative | Positive | 131 | Positive | 5-May-20 | 18.65 |  |
| 57 | 18-Oct-20 | Positive | Positive | 130 | Positive |  |  |  |
| 58 | 19-Oct-20 | Negative | Positive | 130 | Positive | 11-Aug-20 | 17.67 |  |
| 59 | 19-Oct-20 | Negative | Positive | 130 | Positive | 6-May-20 | 33.93 | Moderate |
| 60 | 13-Oct-20 | Negative | Positive | 129 | Positive |  |  |  |
| 61 | 19-Oct-20 | Negative | Positive | 129 | Positive | 22-Jul-20 | 18.05 |  |
| 62 | 19-Oct-20 | Negative | Positive | 129 | Positive | 30-May-20 | 22.79 |  |
| 63 | 18-Oct-20 | Negative | Positive | 129 | Positive | 8-May-20 | 26.6 |  |
| 64 | 19-Oct-20 | Negative | Positive | 128 | Positive |  |  |  |
| 65 | 18-Oct-20 | Positive | Positive | 128 | Positive | 3-Aug-20 | 17.23 |  |
| 66 | 19-Oct-20 | Negative | Positive | 128 | Positive | 19-Jun-20 | 18.71 |  |
| 67 | 19-Oct-20 | Negative | Positive | 128 | Positive | 13-May-20 | 15.64 |  |
| 68 | 18-Oct-20 | Negative | Positive | 127 | Positive |  |  |  |
| 69 | 19-Oct-20 | Negative | Positive | 127 | Positive |  |  |  |
| 70 | 19-Oct-20 | Negative | Positive | 127 | Positive |  |  |  |
| 71 | 19-Oct-20 | Positive | Positive | 127 | Positive | 13-May-20 | 22.14 |  |
| 72 | 17-Oct-20 | Negative | Positive | 126 | Positive |  |  |  |
| 73 | 17-Oct-20 | Negative | Positive | 126 | Positive |  |  |  |
| 74 | 18-Oct-20 | Negative | Positive | 126 | Positive |  |  |  |
| 75 | 19-Oct-20 | Negative | Positive | 126 | Positive | 20-Jul-20 | 23.21 | Moderate |
| 76 | 19-Oct-20 | Negative | Positive | 126 | Positive | 23-Jun-20 | 18.79 |  |
| 77 | 18-Oct-20 | Negative | Positive | 125 | Positive | 10-Aug-20 | 16.09 |  |
| 78 | 18-Oct-20 | Positive | Positive | 125 | Positive | 17-Jun-20 | 18.8 |  |
| 79 | 19-Oct-20 | Negative | Positive | 125 | Positive | 7-Jun-20 | 21.79 |  |
| 80 | 17-Oct-20 | Negative | Positive | 125 | Positive | 1-Jun-20 | 17.26 |  |
| 81 | 18-Oct-20 | Negative | Positive | 124 | Positive |  |  |  |
| 82 | 18-Oct-20 | Negative | Positive | 124 | Positive |  |  |  |
| 83 | 18-Oct-20 | Negative | Positive | 123 | Positive |  |  |  |
| 84 | 19-Oct-20 | Positive | Positive | 123 | Positive | 8-Aug-20 | 15.49 |  |
| 85 | 12-Oct-20 | Negative | Positive | 123 | Positive | 28-Jun-20 | 17.15 |  |
| 86 | 18-Oct-20 | Negative | Positive | 123 | Positive | 14-May-20 | 26.36 |  |
| 87 | 18-Oct-20 | Negative | Positive | 122 | Positive |  |  |  |
| 88 | 17-Oct-20 | Negative | Positive | 122 | Positive |  |  |  |
| 89 | 19-Oct-20 | Negative | Positive | 122 | Positive |  |  |  |
| 90 | 19-Oct-20 | Negative | Positive | 122 | Positive | 2-Jun-20 | 22.47 |  |
| 91 | 10-Oct-20 | Negative | Positive | 122 | Positive | 10-May-20 | 21.19 |  |
| 92 | 13-Oct-20 | Negative | Positive | 121 | Positive |  |  |  |
| 93 | 18-Oct-20 | Negative | Positive | 121 | Positive |  |  |  |
| 94 | 18-Oct-20 | Negative | Positive | 121 | Positive |  |  |  |
| 95 | 19-Oct-20 | Negative | Positive | 121 | Positive | 11-Aug-20 | 19.61 |  |
| 96 | 13-Oct-20 | Negative | Positive | 120 | Positive | 29-Apr-20 | 17 |  |
| 97 | 18-Oct-20 | Negative | Positive | 119 | Positive |  |  |  |
| 98 | 18-Oct-20 | Negative | Positive | 119 | Positive |  |  |  |
| 99 | 18-Oct-20 | Negative | Positive | 119 | Positive |  |  |  |
| 100 | 11-Oct-20 | Negative | Positive | 119 | Positive | 28-May-20 | 17.58 |  |
| 101 | 19-Oct-20 | Negative | Positive | 119 | Positive | 1-Jun-20 | 29.04 |  |
| 102 | 18-Oct-20 | Positive | Positive | 119 | Positive | 9-May-20 | 25.23 | Critical |
| 103 | 19-Oct-20 | Negative | Positive | 118 | Positive |  |  |  |
| 104 | 19-Oct-20 | Negative | Positive | 118 | Positive |  |  |  |
| 105 | 14-Oct-20 | Negative | Positive | 118 | Positive | 2-Jul-20 | 20.16 |  |
| 106 | 18-Oct-20 | Positive | Positive | 118 | Positive | 26-Jun-20 | 19.85 |  |
| 107 | 18-Oct-20 | Negative | Positive | 118 | Positive | 21-May-20 |  |  |
| 108 | 19-Oct-20 | Negative | Positive | 117 | Positive |  |  |  |
| 109 | 19-Oct-20 | Negative | Positive | 117 | Positive | 24-May-20 | 24.01 |  |
| 110 | 18-Oct-20 | Positive | Positive | 117 | Positive | 9-May-20 | 27.86 |  |
| 111 | 17-Oct-20 | Negative | Positive | 116 | Positive |  |  |  |
| 112 | 19-Oct-20 | Negative | Positive | 116 | Positive |  |  |  |
| 113 | 19-Oct-20 | Negative | Positive | 116 | Positive | 17-Jun-20 | 22 | Critical |
| 114 | 12-Oct-20 | Positive | Positive | 115 | Positive | 6-Aug-20 | 24.09 |  |
| 115 | 18-Oct-20 | Negative | Positive | 114 | Positive |  |  |  |
| 116 | 18-Oct-20 | Negative | Positive | 114 | Positive |  |  |  |
| 117 | 18-Oct-20 | Negative | Positive | 114 | Positive | 9-Jul-20 | 18.76 |  |
| 118 | 18-Oct-20 | Negative | Positive | 114 | Positive | 6-May-20 | 26.72 | Mild |
| 119 | 14-Oct-20 | Negative | Positive | 114 | Positive | 22-Apr-20 | 15.56 |  |
| 120 | 18-Oct-20 | Negative | Positive | 113 | Positive |  |  |  |
| 121 | 15-Oct-20 | Negative | Positive | 113 | Positive |  |  |  |
| 122 | 19-Oct-20 | Negative | Positive | 113 | Positive |  |  |  |
| 123 | 18-Oct-20 | Negative | Positive | 113 | Positive | 29-Jun-20 | 16.83 | Severe |
| 124 | 15-Oct-20 | Negative | Positive | 113 | Positive | 2-May-20 | 17.93 |  |
| 125 | 18-Oct-20 | Negative | Positive | 111 | Positive |  |  |  |
| 126 | 18-Oct-20 | Negative | Positive | 111 | Positive |  |  |  |
| 127 | 19-Oct-20 | Negative | Positive | 111 | Positive |  |  |  |
| 128 | 12-Oct-20 | Negative | Positive | 111 | Positive |  |  |  |
| 129 | 19-Oct-20 | Negative | Positive | 111 | Positive |  |  |  |
| 130 | 18-Oct-20 | Negative | Positive | 111 | Positive | 9-Aug-20 | 20.45 | Severe |
| 131 | 19-Oct-20 | Positive | Positive | 111 | Positive | 25-Jun-20 | 15.48 | Moderate |
| 132 | 18-Oct-20 | Negative | Positive | 111 | Positive | 10-May-20 | 34.51 |  |
| 133 | 18-Oct-20 | Negative | Positive | 111 | Positive | 9-May-20 | 24.82 |  |
| 134 | 12-Oct-20 | Negative | Positive | 110 | Positive |  |  |  |
| 135 | 19-Oct-20 | Negative | Negative | 110 | Positive |  |  |  |
| 136 | 19-Oct-20 | Negative | Positive | 110 | Positive | 14-Aug-20 | 20.71 | Moderate |
| 137 | 16-Oct-20 | Negative | Positive | 110 | Positive | 11-Aug-20 | 21.98 |  |
| 138 | 13-Oct-20 | Negative | Positive | 108 | Positive | 1-May-20 | 17.94 |  |
| 139 | 16-Oct-20 | Negative | Positive | 107 | Positive |  |  |  |
| 140 | 12-Oct-20 | Negative | Positive | 107 | Positive | 12-Apr-20 | 17.95 |  |
| 141 | 11-Oct-20 | Negative | Positive | 106 | Positive |  |  |  |
| 142 | 18-Oct-20 | Positive | Positive | 106 | Positive |  |  |  |
| 143 | 18-Oct-20 | Negative | Positive | 106 | Positive | 15-Oct-20 | 34.43 |  |
| 144 | 11-Oct-20 | Negative | Positive | 105 | Positive |  |  |  |
| 145 | 19-Oct-20 | Negative | Positive | 105 | Positive |  |  |  |
| 146 | 19-Oct-20 | Negative | Positive | 105 | Positive | 20-May-20 | 32.54 | Moderate |
| 147 | 16-Oct-20 | Negative | Positive | 104 | Positive |  |  |  |
| 148 | 18-Oct-20 | Negative | Positive | 104 | Positive |  |  |  |
| 149 | 18-Oct-20 | Positive | Positive | 104 | Positive |  |  |  |
| 150 | 19-Oct-20 | Negative | Positive | 104 | Positive | 25-Aug-20 | 13.42 |  |
| 151 | 19-Oct-20 | Negative | Positive | 104 | Positive | 11-Aug-20 | 18.06 |  |
| 152 | 16-Oct-20 | Negative | Positive | 104 | Positive | 18-Jun-20 | 15.55 |  |
| 153 | 19-Oct-20 | Positive | Positive | 104 | Positive | 28-May-20 |  |  |
| 154 | 14-Oct-20 | Positive | Positive | 104 | Positive | 11-May-20 | 26.49 |  |
| 155 | 18-Oct-20 | Negative | Positive | 104 | Positive | 10-May-20 | 30.27 |  |
| 156 | 18-Oct-20 | Negative | Positive | 104 | Positive | 3-May-20 | 23.89 | Critical |
| 157 | 19-Oct-20 | Negative | Positive | 103 | Positive |  |  |  |
| 158 | 18-Oct-20 | Negative | Positive | 103 | Positive |  |  |  |
| 159 | 18-Oct-20 | Negative | Positive | 103 | Positive | 31-Aug-20 | 19.3 | Severe |
| 160 | 18-Oct-20 | Negative | Positive | 102 | Positive |  |  |  |
| 161 | 11-Oct-20 | Negative | Positive | 102 | Positive |  |  |  |
| 162 | 13-Oct-20 | Negative | Positive | 102 | Positive |  |  |  |
| 163 | 12-Oct-20 | Negative | Positive | 102 | Positive |  |  |  |
| 164 | 12-Oct-20 | Negative | Positive | 101 | Positive |  |  |  |
| 165 | 15-Oct-20 | Negative | Positive | 101 | Positive |  |  |  |
| 166 | 17-Oct-20 | Positive | Positive | 101 | Positive |  |  |  |
| 167 | 19-Oct-20 | Negative | Positive | 101 | Positive | 13-Aug-20 | 19.55 | Mild |
| 168 | 19-Oct-20 | Negative | Positive | 101 | Positive | 16-Jun-20 | 19.16 | Critical |
| 169 | 19-Oct-20 | Negative | Positive | 101 | Positive | 1-Jun-20 | 18.45 |  |
| 170 | 18-Oct-20 | Positive | Positive | 101 | Positive | 26-May-20 | 21.34 |  |
| 171 | 19-Oct-20 | Negative | Positive | 101 | Positive | 28-Apr-20 | 19.55 |  |
| 172 | 14-Oct-20 | Positive | Positive | 101 | Positive | 4-Apr-20 |  |  |
| 173 | 17-Oct-20 | Negative | Positive | 100 | Positive | 27-Jun-20 | 27.77 |  |
| 174 | 17-Oct-20 | Negative | Positive | 98.5 | Positive | 30-Jul-20 | 33.92 |  |
| 175 | 16-Oct-20 | Negative | Positive | 97.6 | Positive |  |  |  |
| 176 | 15-Oct-20 | Negative | Positive | 97.5 | Positive |  |  |  |
| 177 | 18-Oct-20 | Negative | Positive | 97.3 | Positive | 8-May-20 | 18.57 |  |
| 178 | 14-Oct-20 | Negative | Positive | 96.9 | Positive | 18-Aug-20 | 20.17 |  |
| 179 | 18-Oct-20 | Negative | Positive | 96.9 | Positive | 8-May-20 | 22.26 |  |
| 180 | 15-Oct-20 | Negative | Positive | 96.9 | Positive | 26-Mar-20 |  | Severe |
| 181 | 15-Oct-20 | Negative | Positive | 96.6 | Positive | 1-Jul-20 | 19.86 |  |
| 182 | 19-Oct-20 | Negative | Positive | 96.3 | Positive |  |  |  |
| 183 | 16-Oct-20 | Negative | Negative | 95.6 | Positive |  |  |  |
| 184 | 14-Oct-20 | Negative | Positive | 95 | Positive |  |  |  |
| 185 | 13-Oct-20 | Negative | Positive | 94.2 | Positive |  |  |  |
| 186 | 15-Oct-20 | Negative | Positive | 94.2 | Positive |  |  |  |
| 187 | 13-Oct-20 | Negative | Positive | 93.9 | Positive | 1-May-20 | 28.85 |  |
| 188 | 15-Oct-20 | Negative | Positive | 93.2 | Positive |  |  |  |
| 189 | 12-Oct-20 | Negative | Positive | 93.1 | Positive |  |  |  |
| 190 | 17-Oct-20 | Negative | Positive | 93 | Positive | 1-Jul-20 | 28.65 | Mild |
| 191 | 14-Oct-20 | Positive | Positive | 92.8 | Positive | 30-May-20 | 25.73 |  |
| 192 | 11-Oct-20 | Negative | Positive | 92.1 | Positive | 25-May-20 | 15.64 |  |
| 193 | 15-Oct-20 | Negative | Positive | 92 | Positive | 21-May-20 | 19.92 |  |
| 194 | 12-Oct-20 | Negative | Positive | 91.9 | Positive | 16-Aug-20 | 17.49 |  |
| 195 | 19-Oct-20 | Negative | Positive | 91.5 | Positive |  |  |  |
| 196 | 15-Oct-20 | Negative | Positive | 91.3 | Positive | 12-Aug-20 | 31.95 |  |
| 197 | 18-Oct-20 | Negative | Positive | 90.8 | Positive |  |  |  |
| 198 | 19-Oct-20 | Negative | Positive | 90.4 | Positive |  |  |  |
| 199 | 17-Oct-20 | Negative | Positive | 90.1 | Positive | 24-Jun-20 | 32.5 | Severe |
| 200 | 13-Oct-20 | Negative | Positive | 89.2 | Positive |  |  |  |
| 201 | 14-Oct-20 | Negative | Positive | 88.7 | Positive | 25-Jun-20 | 31.75 |  |
| 202 | 17-Oct-20 | Negative | Positive | 87.7 | Positive | 25-Sep-20 | 29.38 | Mild |
| 203 | 13-Oct-20 | Positive | Positive | 87.4 | Positive | 12-May-20 | 25.37 |  |
| 204 | 11-Oct-20 | Negative | Positive | 87 | Positive | 7-Jul-20 | 17.36 | Moderate |
| 205 | 14-Oct-20 | Negative | Positive | 86.8 | Positive | 26-Jul-20 | 16.18 |  |
| 206 | 11-Oct-20 | Negative | Positive | 86.2 | Positive |  |  |  |
| 207 | 19-Oct-20 | Negative | Positive | 84.8 | Positive | 15-Aug-20 | 19.63 |  |
| 208 | 12-Oct-20 | Positive | Positive | 84.1 | Positive | 3-Sep-20 | 36.35 |  |
| 209 | 14-Oct-20 | Negative | Positive | 83 | Positive |  |  |  |
| 210 | 13-Oct-20 | Negative | Positive | 83 | Positive |  |  |  |
| 211 | 15-Oct-20 | Negative | Positive | 82.1 | Positive |  |  |  |
| 212 | 13-Oct-20 | Negative | Positive | 81.3 | Positive | 10-May-20 |  |  |
| 213 | 12-Oct-20 | Negative | Positive | 79.4 | Positive |  |  |  |
| 214 | 13-Oct-20 | Positive | Positive | 78.5 | Positive |  |  |  |
| 215 | 12-Oct-20 | Negative | Positive | 78 | Positive |  |  |  |
| 216 | 12-Oct-20 | Negative | Positive | 75.2 | Positive | 18-Apr-20 | 16.67 |  |
| 217 | 12-Oct-20 | Negative | Positive | 75.1 | Positive | 18-Apr-20 | 26.54 | Mild |
| 218 | 11-Oct-20 | Positive | Positive | 75 | Positive | 1-Jul-20 | 31.05 |  |
| 219 | 11-Oct-20 | Negative | Positive | 74.3 | Positive | 10-May-20 |  |  |
| 220 | 10-Oct-20 | Negative | Positive | 73.3 | Positive | 10-Sep-20 | 22.8 |  |
| 221 | 14-Oct-20 | Negative | Positive | 73.2 | Positive | 16-May-20 | 17.76 |  |
| 222 | 12-Oct-20 | Negative | Positive | 73 | Positive | 27-Apr-20 | 21.19 |  |
| 223 | 10-Oct-20 | Negative | Positive | 72 | Positive | 10-Jul-20 | 19.4 |  |
| 224 | 18-Oct-20 | Negative | Positive | 71.9 | Positive |  |  |  |
| 225 | 15-Oct-20 | Negative | Positive | 71.5 | Positive |  |  |  |
| 226 | 11-Oct-20 | Negative | Positive | 70.3 | Positive |  |  |  |
| 227 | 13-Oct-20 | Negative | Positive | 70.3 | Positive | 13-May-20 | 21.79 |  |
| 228 | 12-Oct-20 | Negative | Positive | 69.4 | Positive | 8-Sep-20 | 15.98 |  |
| 229 | 12-Oct-20 | Negative | Positive | 68.8 | Positive | 22-Apr-20 | 24.92 |  |
| 230 | 17-Oct-20 | Negative | Negative | 68.7 | Positive |  |  |  |
| 231 | 14-Oct-20 | Negative | Positive | 68.7 | Positive | 11-May-20 | 22.87 |  |
| 232 | 11-Oct-20 | Negative | Positive | 67.4 | Positive | 27-May-20 | 26.06 |  |
| 233 | 14-Oct-20 | Negative | Positive | 66.7 | Positive | 13-Sep-20 | 22.15 |  |
| 234 | 13-Oct-20 | Negative | Positive | 65.6 | Positive | 10-May-20 |  |  |
| 235 | 13-Oct-20 | Negative | Positive | 65.4 | Positive | 4-May-20 | 15.74 |  |
| 236 | 12-Oct-20 | Negative | Negative | 64.3 | Positive |  |  |  |
| 237 | 12-Oct-20 | Negative | Positive | 63.4 | Positive |  |  |  |
| 238 | 12-Oct-20 | Positive | Positive | 63.2 | Positive |  |  |  |
| 239 | 18-Oct-20 | Negative | Positive | 63.2 | Positive | 24-Jun-20 | 28.39 |  |
| 240 | 15-Oct-20 | Negative | Positive | 62.7 | Positive |  |  |  |
| 241 | 12-Oct-20 | Negative | Positive | 62.5 | Positive | 25-Apr-20 | 21.81 |  |
| 242 | 12-Oct-20 | Negative | Positive | 61.9 | Positive | 12-May-20 | 33.7 |  |
| 243 | 15-Oct-20 | Negative | Positive | 61.6 | Positive | 25-Jun-20 | 15.78 |  |
| 244 | 12-Oct-20 | Negative | Positive | 61.3 | Positive | 6-Sep-20 | 38.27 |  |
| 245 | 11-Oct-20 | Negative | Positive | 60.3 | Positive | 10-May-20 |  |  |
| 246 | 12-Oct-20 | Negative | Positive | 60.2 | Positive |  |  |  |
| 247 | 13-Oct-20 | Negative | Positive | 59.6 | Positive | 6-May-20 | 16.3 |  |
| 248 | 13-Oct-20 | Negative | Positive | 59.4 | Positive |  |  |  |
| 249 | 13-Oct-20 | Negative | Positive | 59.1 | Positive | 14-Jun-20 | 22.63 |  |
| 250 | 12-Oct-20 | Negative | Positive | 59 | Positive |  |  |  |
| 251 | 19-Oct-20 | Negative | Positive | 59 | Positive |  |  |  |
| 252 | 19-Oct-20 | Negative | Positive | 56.8 | Positive |  |  |  |
| 253 | 10-Oct-20 | Negative | Positive | 56.7 | Positive |  |  |  |
| 254 | 18-Oct-20 | Negative | Positive | 56.5 | Positive |  |  |  |
| 255 | 12-Oct-20 | Negative | Negative | 55.8 | Positive |  |  |  |
| 256 | 15-Oct-20 | Negative | Positive | 55.6 | Positive |  |  |  |
| 257 | 12-Oct-20 | Negative | Positive | 55.3 | Positive | 9-May-20 | 28.23 |  |
| 258 | 16-Oct-20 | Positive | Positive | 53 | Positive | 2-Jul-20 | 30.14 | Severe |
| 259 | 19-Oct-20 | Negative | Negative | 52.4 | Positive |  |  |  |
| 260 | 12-Oct-20 | Negative | Negative | 51.3 | Positive |  |  |  |
| 261 | 18-Oct-20 | Negative | Positive | 51.1 | Positive |  |  |  |
| 262 | 12-Oct-20 | Negative | Positive | 50.6 | Positive | 13-Jul-20 | 33.1 |  |
| 263 | 18-Oct-20 | Negative | Positive | 49.3 | Positive | 29-Apr-20 | 22.09 |  |
| 264 | 12-Oct-20 | Negative | Positive | 49.2 | Positive |  |  |  |
| 265 | 12-Oct-20 | Negative | Negative | 49.1 | Positive |  |  |  |
| 266 | 14-Oct-20 | Negative | Positive | 49.1 | Positive | 10-May-20 | 30.74 |  |
| 267 | 16-Oct-20 | Negative | Positive | 48.5 | Positive |  |  |  |
| 268 | 12-Oct-20 | Negative | Negative | 48.2 | Positive |  |  |  |
| 269 | 17-Oct-20 | Positive | Positive | 47.4 | Positive | 16-Aug-20 | 27.14 |  |
| 270 | 18-Oct-20 | Negative | Positive | 46.4 | Positive |  |  |  |
| 271 | 18-Oct-20 | Negative | Positive | 46 | Positive |  |  |  |
| 272 | 15-Oct-20 | Negative | Positive | 45.4 | Positive |  |  |  |
| 273 | 17-Oct-20 | Negative | Positive | 45.3 | Positive | 16-Oct-20 | 28.93 |  |
| 274 | 12-Oct-20 | Negative | Positive | 44.5 | Positive | 7-May-20 | 31.93 |  |
| 275 | 15-Oct-20 | Negative | Positive | 44.2 | Positive | 23-May-20 | 22.67 |  |
| 276 | 14-Oct-20 | Negative | Positive | 43.8 | Positive |  |  |  |
| 277 | 12-Oct-20 | Negative | Positive | 43.7 | Positive |  |  |  |
| 278 | 16-Oct-20 | Negative | Positive | 43.4 | Positive |  |  |  |
| 279 | 12-Oct-20 | Negative | Positive | 42.6 | Positive | 20-Jul-20 | 36.28 |  |
| 280 | 15-Oct-20 | Negative | Positive | 42.5 | Positive |  |  |  |
| 281 | 15-Oct-20 | Positive | Positive | 42.4 | Positive | 1-Jun-20 | 19.59 |  |
| 282 | 15-Oct-20 | Negative | Positive | 41.8 | Positive |  |  |  |
| 283 | 17-Oct-20 | Negative | Positive | 41.7 | Positive | 2-Jun-20 | 27.9 |  |
| 284 | 16-Oct-20 | Negative | Positive | 39.5 | Positive | 7-Aug-20 | 22.91 |  |
| 285 | 15-Oct-20 | Negative | Positive | 39.4 | Positive |  |  |  |
| 286 | 14-Oct-20 | Negative | Positive | 38.7 | Positive | 11-May-20 | 33.42 |  |
| 287 | 15-Oct-20 | Negative | Positive | 37.8 | Positive |  |  |  |
| 288 | 15-Oct-20 | Negative | Positive | 37.5 | Positive | 29-May-20 | 22.87 |  |
| 289 | 18-Oct-20 | Negative | Positive | 37.5 | Positive | 8-May-20 | 16.83 |  |
| 290 | 16-Oct-20 | Negative | Positive | 37.1 | Positive |  |  |  |
| 291 | 16-Oct-20 | Negative | Positive | 36.5 | Positive |  |  |  |
| 292 | 15-Oct-20 | Negative | Positive | 35.2 | Positive | 7-Jul-20 | 25.32 |  |
| 293 | 15-Oct-20 | Negative | Positive | 34.8 | Positive | 21-May-20 | 21.06 |  |
| 294 | 15-Oct-20 | Positive | Positive | 34.1 | Positive |  |  |  |
| 295 | 12-Oct-20 | Negative | Positive | 34 | Positive | 3-Sep-20 | 30.04 |  |
| 296 | 19-Oct-20 | Negative | Positive | 33.3 | Positive | 28-Jun-20 | 17.36 |  |
| 297 | 12-Oct-20 | Negative | Positive | 32.7 | Positive | 28-Apr-20 | 17.18 |  |
| 298 | 15-Oct-20 | Negative | Positive | 32.3 | Positive |  |  |  |
| 299 | 14-Oct-20 | Negative | Positive | 31.9 | Positive | 30-Mar-20 |  |  |
| 300 | 11-Oct-20 | Negative | Positive | 31.7 | Positive | 10-May-20 | 32.27 |  |
| 301 | 15-Oct-20 | Negative | Positive | 31.2 | Positive | 22-Jun-20 | 25.95 |  |
| 302 | 12-Oct-20 | Negative | Positive | 30.8 | Positive | 3-Sep-20 | 30.41 |  |
| 303 | 12-Oct-20 | Negative | Positive | 30.3 | Positive | 3-Sep-20 | 30.3 |  |
| 304 | 12-Oct-20 | Negative | Positive | 30.2 | Positive | 26-Apr-20 | 19.84 | Moderate |
| 305 | 17-Oct-20 | Negative | Positive | 29 | Positive |  |  |  |
| 306 | 15-Oct-20 | Negative | Positive | 29 | Positive |  |  |  |
| 307 | 15-Oct-20 | Negative | Negative | 27.9 | Positive | 3-Jun-20 | 22.24 |  |
| 308 | 17-Oct-20 | Negative | Positive | 27.5 | Positive |  |  |  |
| 309 | 19-Oct-20 | Negative | Positive | 27.2 | Positive |  |  |  |
| 310 | 18-Oct-20 | Positive | Positive | 27.2 | Positive | 27-Sep-20 | 18.95 |  |
| 311 | 17-Oct-20 | Negative | Positive | 27.1 | Positive |  |  |  |
| 312 | 13-Oct-20 | Negative | Positive | 27.1 | Positive | 29-Apr-20 | 26.87 |  |
| 313 | 12-Oct-20 | Negative | Positive | 26.2 | Positive | 2-Apr-20 | 32.05 | Mild |
| 314 | 14-Oct-20 | Negative | Positive | 25.7 | Positive |  |  |  |
| 315 | 15-Oct-20 | Negative | Positive | 25.4 | Positive |  |  |  |
| 316 | 18-Oct-20 | Negative | Negative | 25.1 | Positive | 9-May-20 | 34.39 |  |
| 317 | 12-Oct-20 | Negative | Positive | 24.6 | Positive |  |  |  |
| 318 | 19-Oct-20 | Negative | Positive | 24.5 | Positive |  |  |  |
| 319 | 15-Oct-20 | Negative | Positive | 24.3 | Positive | 9-Sep-20 | 20.95 |  |
| 320 | 15-Oct-20 | Negative | Positive | 23.6 | Positive |  |  |  |
| 321 | 15-Oct-20 | Negative | Positive | 23.3 | Positive |  |  |  |
| 322 | 14-Oct-20 | Negative | Positive | 23 | Positive | 16-Jul-20 | 15.98 |  |
| 323 | 15-Oct-20 | Negative | Positive | 22.1 | Positive |  |  |  |
| 324 | 19-Oct-20 | Negative | Positive | 22 | Positive | 26-Sep-20 | 23.97 | Moderate |
| 325 | 12-Oct-20 | Negative | Positive | 21.4 | Positive | 12-May-20 | 21.1 |  |
| 326 | 15-Oct-20 | Negative | Positive | 21.2 | Positive |  |  |  |
| 327 | 14-Oct-20 | Negative | Negative | 21.2 | Positive |  |  |  |
| 328 | 12-Oct-20 | Negative | Positive | 20.7 | Positive | 3-Sep-20 | 27.53 |  |
| 329 | 14-Oct-20 | Positive | Positive | 20.6 | Positive |  |  |  |
| 330 | 14-Oct-20 | Negative | Positive | 20 | Positive | 16-May-20 | 19.63 |  |
| 331 | 18-Oct-20 | Negative | Positive | 19.6 | Positive |  |  |  |
| 332 | 16-Oct-20 | Positive | Positive | 19.6 | Positive | 4-Oct-20 | 16.12 | Severe |
| 333 | 16-Oct-20 | Positive | Negative | 19.4 | Positive | 1-May-20 | 36.79 | Moderate |
| 334 | 18-Oct-20 | Negative | Negative | 19.3 | Positive |  |  |  |
| 335 | 18-Oct-20 | Negative | Positive | 19.2 | Positive |  |  |  |
| 336 | 18-Oct-20 | Negative | Negative | 19.1 | Positive | 27-May-20 | 22.39 |  |
| 337 | 11-Oct-20 | Negative | Negative | 18.6 | Positive | 10-May-20 | 15.92 |  |
| 338 | 14-Oct-20 | Positive | Positive | 18.5 | Positive | 11-May-20 | 15.94 |  |
| 339 | 17-Oct-20 | Negative | Positive | 18.3 | Positive |  |  |  |
| 340 | 19-Oct-20 | Negative | Negative | 18 | Positive | 1-Jul-20 | 17.98 |  |
| 341 | 18-Oct-20 | Negative | Positive | 17.8 | Positive | 1-May-20 | 18.25 |  |
| 342 | 18-Oct-20 | Negative | Positive | 17.7 | Positive | 30-Aug-20 | 28.92 | Critical |
| 343 | 15-Oct-20 | Negative | Positive | 17.5 | Positive | 27-Sep-20 | 19.9 |  |
| 344 | 19-Oct-20 | Negative | Positive | 17.5 | Positive | 4-Apr-20 |  |  |
| 345 | 19-Oct-20 | Negative | Positive | 17.3 | Positive | 25-May-20 | 21.58 |  |
| 346 | 15-Oct-20 | Negative | Positive | 17 | Positive |  |  |  |
| 347 | 12-Oct-20 | Negative | Negative | 16.9 | Positive |  |  |  |
| 348 | 18-Oct-20 | Negative | Negative | 16.8 | Positive |  |  |  |
| 349 | 17-Oct-20 | Positive | Positive | 16.7 | Positive | 7-Jun-20 | 26.25 |  |
| 350 | 18-Oct-20 | Negative | Positive | 16.4 | Positive |  |  |  |
| 351 | 19-Oct-20 | Negative | Positive | 16.3 | Positive |  |  |  |
| 352 | 15-Oct-20 | Negative | Positive | 16.1 | Positive |  |  |  |
| 353 | 13-Oct-20 | Negative | Positive | 16.1 | Positive | 6-May-20 | 33.66 |  |
| 354 | 18-Oct-20 | Negative | Positive | 16 | Positive |  |  |  |
| 355 | 15-Oct-20 | Negative | Positive | 16 | Positive |  |  |  |
| 356 | 19-Oct-20 | Positive | Positive | 15.8 | Positive | 28-Aug-20 | 21.96 |  |
| 357 | 11-Oct-20 | Negative | Positive | 15.8 | Positive | 17-Jun-20 | 19.76 |  |
| 358 | 18-Oct-20 | Negative | Positive | 15.7 | Positive |  |  |  |
| 359 | 18-Oct-20 | Negative | Positive | 15.6 | Positive |  |  |  |
| 360 | 18-Oct-20 | Negative | Positive | 15.2 | Positive | 20-Aug-20 | 14.43 |  |
| 361 | 18-Oct-20 | Positive | Positive | 15.1 | Positive | 28-Sep-20 | 29.58 |  |
| 362 | 18-Oct-20 | Negative | Positive | 15 | Positive | 14-May-20 | 14.87 |  |
| 363 | 15-Oct-20 | Positive | Positive | 14.9 | Positive |  |  |  |
| 364 | 16-Oct-20 | Negative | Positive | 14.8 | Positive |  |  |  |
| 365 | 18-Oct-20 | Negative | Positive | 14.6 | Positive |  |  |  |
| 366 | 15-Oct-20 | Negative | Negative | 14.6 | Positive |  |  |  |
| 367 | 15-Oct-20 | Negative | Positive | 14.5 | Positive |  |  |  |
| 368 | 18-Oct-20 | Positive | Positive | 14.4 | Positive | 8-Oct-20 | 20.75 | Severe |
| 369 | 18-Oct-20 | Negative | Positive | 14.3 | Positive |  |  |  |
| 370 | 18-Oct-20 | Negative | Positive | 14.3 | Positive |  |  |  |
| 371 | 19-Oct-20 | Negative | Positive | 14.3 | Positive |  |  |  |
| 372 | 19-Oct-20 | Negative | Negative | 14.3 | Positive | 28-May-20 | 30.7 | Critical |
| 373 | 19-Oct-20 | Negative | Positive | 14.3 | Positive | 21-Apr-20 | 24.38 |  |
| 374 | 19-Oct-20 | Negative | Positive | 14.2 | Positive | 11-Aug-20 | 15.83 |  |
| 375 | 19-Oct-20 | Negative | Positive | 14.2 | Positive | 21-May-20 | 16.53 |  |
| 376 | 12-Oct-20 | Negative | Negative | 14.1 | Positive |  |  |  |
| 377 | 17-Oct-20 | Negative | Positive | 14 | Positive |  |  |  |
| 378 | 19-Oct-20 | Negative | Positive | 13.9 | Positive |  |  |  |
| 379 | 19-Oct-20 | Negative | Positive | 13.9 | Positive | 5-Jun-20 | 23.94 |  |
| 380 | 18-Oct-20 | Negative | Negative | 13.6 | Positive |  |  |  |
| 381 | 19-Oct-20 | Negative | Positive | 13.5 | Positive |  |  |  |
| 382 | 18-Oct-20 | Negative | Positive | 13.4 | Positive |  |  |  |
| 383 | 15-Oct-20 | Negative | Positive | 13.4 | Positive |  |  |  |
| 384 | 17-Oct-20 | Negative | Positive | 13.4 | Positive | 12-Oct-20 | 24.38 |  |
| 385 | 12-Oct-20 | Positive | Positive | 13.4 | Positive | 22-Aug-20 | 23.82 |  |
| 386 | 19-Oct-20 | Negative | Positive | 13.4 | Positive | 29-Jul-20 | 28.09 |  |
| 387 | 19-Oct-20 | Negative | Positive | 13.4 | Positive | 30-Jun-20 | 15.8 |  |
| 388 | 19-Oct-20 | Negative | Positive | 13.3 | Positive |  |  |  |
| 389 | 19-Oct-20 | Negative | Positive | 13.2 | Positive | 25-May-20 | 30.72 |  |
| 390 | 19-Oct-20 | Negative | Positive | 13.1 | Positive |  |  |  |
| 391 | 18-Oct-20 | Negative | Positive | 13 | Positive |  |  |  |
| 392 | 15-Oct-20 | Negative | Positive | 12.9 | Positive |  |  |  |
| 393 | 19-Oct-20 | Negative | Positive | 12.9 | Positive |  |  |  |
| 394 | 17-Oct-20 | Negative | Positive | 12.9 | Positive | 3-Oct-20 | 15.44 | Moderate |
| 395 | 19-Oct-20 | Negative | Positive | 12.8 | Positive | 17-Aug-20 | 29.98 |  |
| 396 | 12-Oct-20 | Negative | Positive | 12.7 | Positive |  |  |  |
| 397 | 19-Oct-20 | Negative | Positive | 12.6 | Positive |  |  |  |
| 398 | 18-Oct-20 | Negative | Positive | 12.6 | Positive |  |  |  |
| 399 | 16-Oct-20 | Negative | Negative | 12.6 | Positive |  |  |  |
| 400 | 19-Oct-20 | Negative | Positive | 12.5 | Positive |  |  |  |
| 401 | 19-Oct-20 | Negative | Positive | 12.5 | Positive | 3-Jul-20 | 17.87 |  |
| 402 | 19-Oct-20 | Negative | Positive | 12.4 | Positive |  |  |  |
| 403 | 19-Oct-20 | Negative | Positive | 12.4 | Positive | 1-Oct-20 | 17.72 |  |
| 404 | 15-Oct-20 | Negative | Negative | 12.3 | Positive |  |  |  |
| 405 | 11-Oct-20 | Negative | Positive | 12.2 | Positive |  |  |  |
| 406 | 15-Oct-20 | Negative | Negative | 12.2 | Positive |  |  |  |
| 407 | 19-Oct-20 | Negative | Negative | 12.2 | Positive |  |  |  |
| 408 | 19-Oct-20 | Negative | Positive | 11.9 | Positive |  |  |  |
| 409 | 19-Oct-20 | Negative | Positive | 11.9 | Positive |  |  |  |
| 410 | 15-Oct-20 | Negative | Positive | 11.7 | Positive |  |  |  |
| 411 | 16-Oct-20 | Negative | Negative | 11.7 | Positive |  |  |  |
| 412 | 19-Oct-20 | Negative | Positive | 11.6 | Positive |  |  |  |
| 413 | 19-Oct-20 | Negative | Positive | 11.5 | Positive |  |  |  |
| 414 | 19-Oct-20 | Negative | Positive | 11.4 | Positive |  |  |  |
| 415 | 15-Oct-20 | Negative | Positive | 11.4 | Positive | 4-May-20 | 21.81 |  |
| 416 | 12-Oct-20 | Negative | Negative | 11.3 | Positive |  |  |  |
| 417 | 15-Oct-20 | Negative | Positive | 11.3 | Positive | 8-May-20 | 24.15 |  |
| 418 | 14-Oct-20 | Negative | Positive | 11.3 | Positive | 29-Apr-20 | 31.72 |  |
| 419 | 19-Oct-20 | Negative | Negative | 11.2 | Positive | 26-Jul-20 | 31.9 |  |
| 420 | 18-Oct-20 | Negative | Negative | 10.9 | Positive | 24-May-20 | 35.13 |  |
| 421 | 19-Oct-20 | Negative | Positive | 10.8 | Positive |  |  |  |
| 422 | 19-Oct-20 | Negative | Positive | 10.8 | Positive |  |  |  |
| 423 | 19-Oct-20 | Negative | Positive | 10.8 | Positive |  |  |  |
| 424 | 19-Oct-20 | Negative | Positive | 10.8 | Positive | 16-May-20 | 17.32 |  |
| 425 | 13-Oct-20 | Negative | Negative | 10.8 | Positive | 29-Apr-20 | 16.22 |  |
| 426 | 12-Oct-20 | Negative | Negative | 10.3 | Positive |  |  |  |
| 427 | 19-Oct-20 | Negative | Positive | 10.3 | Positive | 2-Jun-20 |  |  |
| 428 | 19-Oct-20 | Negative | Positive | 10.2 | Positive |  |  |  |
| 429 | 18-Oct-20 | Negative | Negative | 10 | Positive | 7-Jun-20 | 32 |  |
| 430 | 12-Oct-20 | Positive | Positive | 9.81 | Positive | 20-Sep-20 | 16.94 |  |
| 431 | 16-Oct-20 | Negative | Positive | 9.7 | Positive |  |  |  |
| 432 | 16-Oct-20 | Positive | Positive | 9.53 | Positive | 15-Oct-20 | 28.73 |  |
| 433 | 16-Oct-20 | Negative | Positive | 9.46 | Positive |  |  |  |
| 434 | 15-Oct-20 | Negative | Positive | 9.45 | Positive | 21-Sep-20 | 23.69 |  |
| 435 | 13-Oct-20 | Negative | Positive | 9.28 | Positive | 28-Apr-20 | 28.63 |  |
| 436 | 18-Oct-20 | Negative | Positive | 9.24 | Positive |  |  |  |
| 437 | 17-Oct-20 | Negative | Negative | 9.23 | Positive |  |  |  |
| 438 | 17-Oct-20 | Negative | Positive | 9.15 | Positive | 11-Aug-20 | 19.86 |  |
| 439 | 16-Oct-20 | Negative | Positive | 9.12 | Positive |  |  |  |
| 440 | 16-Oct-20 | Negative | Positive | 9.06 | Positive | 14-Oct-20 | 21.97 |  |
| 441 | 17-Oct-20 | Negative | Negative | 8.99 | Positive |  |  |  |
| 442 | 13-Oct-20 | Negative | Positive | 8.96 | Positive | 12-May-20 | 20.85 |  |
| 443 | 15-Oct-20 | Negative | Positive | 8.69 | Positive | 27-Sep-20 | 24.92 |  |
| 444 | 16-Oct-20 | Positive | Positive | 8.5 | Positive | 30-Jul-20 | 26.75 | Severe |
| 445 | 17-Oct-20 | Positive | Positive | 8.4 | Positive | 14-Oct-20 | 33.43 | Severe |
| 446 | 13-Oct-20 | Negative | Positive | 8.1 | Positive | 28-Apr-20 | 31.96 |  |
| 447 | 17-Oct-20 | Positive | Negative | 8.01 | Positive |  |  |  |
| 448 | 17-Oct-20 | Negative | Positive | 7.52 | Positive |  |  |  |
| 449 | 12-Oct-20 | Negative | Negative | 7.28 | Positive |  |  |  |
| 450 | 17-Oct-20 | Negative | Positive | 7.2 | Positive | 13-Oct-20 | 28.61 | Moderate |
| 451 | 12-Oct-20 | Negative | Negative | 7.06 | Positive |  |  |  |
| 452 | 17-Oct-20 | Negative | Negative | 6.82 | Positive |  |  |  |
| 453 | 15-Oct-20 | Negative | Positive | 6.58 | Positive |  |  |  |
| 454 | 17-Oct-20 | Positive | Negative | 6.5 | Positive | 7-Oct-20 | 13.7 | Severe |
| 455 | 18-Oct-20 | Negative | Positive | 6.48 | Positive | 18-Sep-20 | 29.08 |  |
| 456 | 14-Oct-20 | Negative | Positive | 6.29 | Positive |  |  |  |
| 457 | 17-Oct-20 | Negative | Positive | 5.64 | Positive | 28-May-20 | 35.02 |  |
| 458 | 10-Oct-20 | Negative | Positive | 5.57 | Positive | 21-Sep-20 | 16.41 |  |
| 459 | 18-Oct-20 | Positive | Positive | 5.42 | Positive |  |  |  |
| 460 | 17-Oct-20 | Positive | Positive | 5.31 | Positive | 16-Oct-20 | 21.72 | Severe |
| 461 | 13-Oct-20 | Negative | Negative | 5.3 | Positive | 28-Apr-20 | 36.08 |  |
| 462 | 16-Oct-20 | Negative | Positive | 5.28 | Positive |  |  |  |
| 463 | 18-Oct-20 | Negative | Positive | 5.04 | Positive |  |  |  |
| 464 | 10-Oct-20 | Negative | Negative | 4.94 | Positive | 21-Sep-20 | 28.08 |  |
| 465 | 13-Oct-20 | Negative | Positive | 4.9 | Positive | 6-May-20 | 20.53 |  |
| 466 | 17-Oct-20 | Negative | Positive | 4.84 | Positive | 4-Oct-20 | 17.48 | Mild |
| 467 | 10-Oct-20 | Positive | Positive | 4.83 | Positive | 22-Sep-20 | 29.53 |  |
| 468 | 12-Oct-20 | Negative | Positive | 4.78 | Positive |  |  |  |
| 469 | 15-Oct-20 | Negative | Positive | 4.75 | Positive |  |  |  |
| 470 | 17-Oct-20 | Negative | Negative | 4.7 | Positive | 13-Oct-20 | 19.41 | Moderate |
| 471 | 13-Oct-20 | Negative | Negative | 4.53 | Positive | 10-May-20 |  |  |
| 472 | 15-Oct-20 | Negative | Negative | 4.47 | Positive |  |  |  |
| 473 | 17-Oct-20 | Negative | Negative | 4.36 | Positive |  |  |  |
| 474 | 17-Oct-20 | Negative | Negative | 4.29 | Positive | 11-Oct-20 | 23.26 |  |
| 475 | 17-Oct-20 | Negative | Positive | 4.16 | Positive | 16-Oct-20 | 35.71 |  |
| 476 | 17-Oct-20 | Negative | Positive | 4.14 | Positive | 13-Jun-20 | 16.94 |  |
| 477 | 10-Oct-20 | Negative | Positive | 4.1 | Positive | 19-Sep-20 | 19.17 |  |
| 478 | 16-Oct-20 | Negative | Positive | 3.94 | Positive | 15-Oct-20 | 29 |  |
| 479 | 17-Oct-20 | Positive | Positive | 3.63 | Positive | 15-Oct-20 | 26.61 |  |
| 480 | 13-Oct-20 | Negative | Positive | 3.6 | Positive | 28-Apr-20 | 15.24 |  |
| 481 | 14-Oct-20 | Negative | Positive | 3.5 | Positive | 17-Aug-20 | 12.5 |  |
| 482 | 19-Oct-20 | Negative | Positive | 3.32 | Positive | 24-Jun-20 | 21.62 |  |
| 483 | 17-Oct-20 | Negative | Positive | 3.19 | Positive |  |  |  |
| 484 | 10-Oct-20 | Negative | Negative | 3.16 | Positive | 22-Sep-20 | 19.1 |  |
| 485 | 18-Oct-20 | Negative | Negative | 3.15 | Positive |  |  |  |
| 486 | 16-Oct-20 | Negative | Negative | 3.14 | Positive |  |  |  |
| 487 | 16-Oct-20 | Negative | Positive | 3 | Positive | 6-Oct-20 | 15.29 |  |
| 488 | 13-Oct-20 | Negative | Negative | 2.95 | Positive | 28-Apr-20 | 17.55 |  |
| 489 | 16-Oct-20 | Negative | Positive | 2.62 | Positive | 20-Sep-20 | 15.9 |  |
| 490 | 15-Oct-20 | Negative | Positive | 2.59 | Positive | 11-Oct-20 | 18.55 |  |
| 491 | 19-Oct-20 | Negative | Positive | 2.45 | Positive |  |  |  |
| 492 | 17-Oct-20 | Positive | Positive | 2.31 | Positive | 29-Sep-20 | 32.58 |  |
| 493 | 15-Oct-20 | Negative | Positive | 2.23 | Positive |  |  |  |
| 494 | 16-Oct-20 | Negative | Positive | 2.2 | Positive | 4-Oct-20 | 23.7 | Severe |
| 495 | 15-Oct-20 | Negative | Positive | 2.18 | Positive |  |  |  |
| 496 | 18-Oct-20 | Positive | Positive | 2.11 | Positive |  |  |  |
| 497 | 18-Oct-20 | Negative | Negative | 1.99 | Positive | 27-May-20 | 32.17 |  |
| 498 | 12-Oct-20 | Negative | Negative | 1.63 | Positive |  |  |  |
| 499 | 17-Oct-20 | Negative | Negative | 1.58 | Positive |  |  |  |
| 500 | 19-Oct-20 | Negative | Positive | 1.57 | Positive | 9-Aug-20 | 20.25 |  |
| 501 | 18-Oct-20 | Negative | Positive | 1.54 | Positive | 29-Sep-20 | 22.78 |  |
| 502 | 18-Oct-20 | Negative | Negative | 1.49 | Positive |  |  |  |
| 503 | 16-Oct-20 | Negative | Negative | 1.47 | Positive |  |  |  |
| 504 | 18-Oct-20 | Negative | Positive | 1.47 | Positive | 20-May-20 | 22.9 | Critical |
| 505 | 18-Oct-20 | Negative | Negative | 1.45 | Positive |  |  |  |
| 506 | 16-Oct-20 | Positive | Positive | 1.42 | Positive | 13-Oct-20 | 28.76 | Moderate |
| 507 | 17-Oct-20 | Negative | Positive | 1.24 | Positive |  |  |  |
| 508 | 10-Oct-20 | Negative | Positive | 1.18 | Positive | 19-Sep-20 | 17.55 |  |
| 509 | 19-Oct-20 | Negative | Negative | 1.1 | Positive | 27-Oct-20 | 22.94 |  |
| 510 | 10-Oct-20 | Negative | Positive | 1.01 | Positive |  |  |  |
| 511 | 17-Oct-20 | Negative | Negative | 0.66 | Negative | 13-Oct-20 | 15.64 | Moderate |
| 512 | 19-Oct-20 | Negative | Negative | 0.11 | Negative |  |  |  |
| 513 | 19-Oct-20 | Negative | Negative | 0.1 | Negative |  |  |  |
| 514 | 19-Oct-20 | Negative | Negative | 0.1 | Negative |  |  |  |
| 515 | 19-Oct-20 | Negative | Negative | 0.1 | Negative |  |  |  |
| 516 | 19-Oct-20 | Negative | Negative | 0.1 | Negative |  |  |  |
| 517 | 19-Oct-20 | Negative | Negative | 0.1 | Negative |  |  |  |
| 518 | 19-Oct-20 | Negative | Negative | 0.1 | Negative |  |  |  |
| 519 | 19-Oct-20 | Negative | Negative | 0.1 | Negative |  |  |  |
| 520 | 19-Oct-20 | Negative | Negative | 0.1 | Negative |  |  |  |
| 521 | 21-Oct-20 | Negative | Negative | 0.1 | Negative |  |  |  |
| 522 | 19-Oct-20 | Negative | Negative | 0.1 | Negative |  |  |  |
| 523 | 19-Oct-20 | Negative | Negative | 0.09 | Negative |  |  |  |
| 524 | 19-Oct-20 | Negative | Negative | 0.09 | Negative |  |  |  |
| 525 | 19-Oct-20 | Negative | Negative | 0.09 | Negative |  |  |  |
| 526 | 19-Oct-20 | Negative | Negative | 0.09 | Negative |  |  |  |
| 527 | 20-Oct-20 | Negative | Negative | 0.09 | Negative |  |  |  |
| 528 | 19-Oct-20 | Negative | Negative | 0.09 | Negative |  |  |  |
| 529 | 19-Oct-20 | Negative | Negative | 0.09 | Negative |  |  |  |
| 530 | 19-Oct-20 | Negative | Negative | 0.09 | Negative |  |  |  |
| 531 | 19-Oct-20 | Negative | Negative | 0.09 | Negative |  |  |  |
| 532 | 20-Oct-20 | Negative | Negative | 0.09 | Negative |  |  |  |
| 533 | 21-Oct-20 | Negative | Negative | 0.09 | Negative |  |  |  |
| 534 | 19-Oct-20 | Negative | Negative | 0.09 | Negative |  |  |  |
| 535 | 21-Oct-20 | Negative | Negative | 0.09 | Negative |  |  |  |
| 536 | 20-Oct-20 | Negative | Negative | 0.09 | Negative |  |  |  |
| 537 | 21-Oct-20 | Negative | Negative | 0.09 | Negative |  |  |  |
| 538 | 20-Oct-20 | Negative | Negative | 0.09 | Negative |  |  |  |
| 539 | 19-Oct-20 | Negative | Negative | 0.09 | Negative |  |  |  |
| 540 | 21-Oct-20 | Negative | Negative | 0.09 | Negative |  |  |  |
| 541 | 19-Oct-20 | Negative | Negative | 0.09 | Negative |  |  |  |
| 542 | 19-Oct-20 | Negative | Negative | 0.09 | Negative |  |  |  |
| 543 | 21-Oct-20 | Negative | Negative | 0.09 | Negative |  |  |  |
| 544 | 21-Oct-20 | Negative | Negative | 0.09 | Negative |  |  |  |
| 545 | 20-Oct-20 | Negative | Negative | 0.09 | Negative |  |  |  |
| 546 | 19-Oct-20 | Negative | Negative | 0.09 | Negative |  |  |  |
| 547 | 19-Oct-20 | Negative | Negative | 0.09 | Negative |  |  |  |
| 548 | 20-Oct-20 | Negative | Negative | 0.09 | Negative |  |  |  |
| 549 | 19-Oct-20 | Negative | Negative | 0.09 | Negative |  |  |  |
| 550 | 19-Oct-20 | Negative | Negative | 0.09 | Negative |  |  |  |
| 551 | 20-Oct-20 | Negative | Negative | 0.09 | Negative |  |  |  |
| 552 | 19-Oct-20 | Negative | Negative | 0.09 | Negative |  |  |  |
| 553 | 21-Oct-20 | Negative | Negative | 0.09 | Negative |  |  |  |
| 554 | 19-Oct-20 | Negative | Negative | 0.09 | Negative |  |  |  |
| 555 | 19-Oct-20 | Negative | Negative | 0.09 | Negative |  |  |  |
| 556 | 19-Oct-20 | Negative | Negative | 0.09 | Negative |  |  |  |
| 557 | 20-Oct-20 | Negative | Negative | 0.09 | Negative |  |  |  |
| 558 | 19-Oct-20 | Negative | Negative | 0.09 | Negative |  |  |  |
| 559 | 19-Oct-20 | Negative | Negative | 0.09 | Negative |  |  |  |
| 560 | 20-Oct-20 | Negative | Negative | 0.09 | Negative |  |  |  |
| 561 | 21-Oct-20 | Negative | Negative | 0.09 | Negative |  |  |  |
| 562 | 20-Oct-20 | Negative | Negative | 0.09 | Negative |  |  |  |
| 563 | 19-Oct-20 | Negative | Negative | 0.09 | Negative |  |  |  |
| 564 | 21-Oct-20 | Negative | Negative | 0.09 | Negative |  |  |  |
| 565 | 19-Oct-20 | Negative | Negative | 0.09 | Negative |  |  |  |
| 566 | 19-Oct-20 | Negative | Negative | 0.09 | Negative |  |  |  |
| 567 | 19-Oct-20 | Negative | Negative | 0.09 | Negative |  |  |  |
| 568 | 19-Oct-20 | Negative | Negative | 0.09 | Negative |  |  |  |
| 569 | 19-Oct-20 | Negative | Negative | 0.09 | Negative |  |  |  |
| 570 | 21-Oct-20 | Negative | Negative | 0.09 | Negative |  |  |  |
| 571 | 21-Oct-20 | Negative | Negative | 0.09 | Negative |  |  |  |
| 572 | 21-Oct-20 | Negative | Negative | 0.09 | Negative |  |  |  |
| 573 | 19-Oct-20 | Negative | Negative | 0.09 | Negative |  |  |  |
| 574 | 19-Oct-20 | Negative | Negative | 0.09 | Negative |  |  |  |
| 575 | 21-Oct-20 | Negative | Negative | 0.09 | Negative |  |  |  |
| 576 | 21-Oct-20 | Negative | Negative | 0.09 | Negative |  |  |  |
| 577 | 21-Oct-20 | Negative | Negative | 0.09 | Negative |  |  |  |
| 578 | 19-Oct-20 | Negative | Negative | 0.09 | Negative |  |  |  |
| 579 | 19-Oct-20 | Negative | Negative | 0.09 | Negative |  |  |  |
| 580 | 21-Oct-20 | Negative | Negative | 0.09 | Negative |  |  |  |
| 581 | 19-Oct-20 | Negative | Negative | 0.09 | Negative |  |  |  |
| 582 | 21-Oct-20 | Negative | Negative | 0.09 | Negative |  |  |  |
| 583 | 19-Oct-20 | Negative | Negative | 0.09 | Negative |  |  |  |
| 584 | 19-Oct-20 | Negative | Negative | 0.09 | Negative |  |  |  |
| 585 | 21-Oct-20 | Negative | Negative | 0.09 | Negative |  |  |  |
| 586 | 21-Oct-20 | Negative | Negative | 0.09 | Negative |  |  |  |
| 587 | 21-Oct-20 | Negative | Negative | 0.09 | Negative |  |  |  |
| 588 | 20-Oct-20 | Negative | Negative | 0.09 | Negative |  |  |  |
| 589 | 19-Oct-20 | Negative | Negative | 0.09 | Negative |  |  |  |
| 590 | 20-Oct-20 | Negative | Negative | 0.09 | Negative |  |  |  |
| 591 | 20-Oct-20 | Negative | Negative | 0.09 | Negative |  |  |  |
| 592 | 19-Oct-20 | Negative | Negative | 0.09 | Negative |  |  |  |
| 593 | 19-Oct-20 | Negative | Negative | 0.09 | Negative |  |  |  |
| 594 | 21-Oct-20 | Negative | Negative | 0.09 | Negative |  |  |  |
| 595 | 19-Oct-20 | Negative | Negative | 0.09 | Negative |  |  |  |
| 596 | 19-Oct-20 | Negative | Negative | 0.09 | Negative |  |  |  |
| 597 | 19-Oct-20 | Negative | Negative | 0.09 | Negative |  |  |  |
| 598 | 19-Oct-20 | Negative | Negative | 0.09 | Negative |  |  |  |
| 599 | 21-Oct-20 | Negative | Negative | 0.09 | Negative |  |  |  |
| 600 | 21-Oct-20 | Negative | Negative | 0.09 | Negative |  |  |  |
| 601 | 19-Oct-20 | Negative | Negative | 0.09 | Negative |  |  |  |
| 602 | 19-Oct-20 | Negative | Negative | 0.09 | Negative |  |  |  |
| 603 | 21-Oct-20 | Negative | Negative | 0.09 | Negative |  |  |  |
| 604 | 20-Oct-20 | Negative | Negative | 0.09 | Negative |  |  |  |
| 605 | 21-Oct-20 | Negative | Negative | 0.09 | Negative |  |  |  |
| 606 | 21-Oct-20 | Negative | Negative | 0.09 | Negative |  |  |  |
| 607 | 21-Oct-20 | Negative | Negative | 0.09 | Negative |  |  |  |
| 608 | 19-Oct-20 | Negative | Negative | 0.09 | Negative |  |  |  |
| 609 | 21-Oct-20 | Negative | Negative | 0.09 | Negative |  |  |  |
| 610 | 19-Oct-20 | Negative | Negative | 0.09 | Negative |  |  |  |
| 611 | 19-Oct-20 | Negative | Negative | 0.09 | Negative |  |  |  |
| 612 | 19-Oct-20 | Negative | Negative | 0.09 | Negative |  |  |  |
| 613 | 20-Oct-20 | Negative | Negative | 0.09 | Negative |  |  |  |
| 614 | 21-Oct-20 | Negative | Negative | 0.09 | Negative |  |  |  |
| 615 | 19-Oct-20 | Negative | Negative | 0.09 | Negative |  |  |  |
| 616 | 21-Oct-20 | Negative | Negative | 0.09 | Negative |  |  |  |
| 617 | 21-Oct-20 | Negative | Negative | 0.09 | Negative |  |  |  |
| 618 | 20-Oct-20 | Negative | Negative | 0.09 | Negative |  |  |  |
| 619 | 21-Oct-20 | Negative | Negative | 0.09 | Negative |  |  |  |
| 620 | 21-Oct-20 | Negative | Negative | 0.09 | Negative |  |  |  |
| 621 | 19-Oct-20 | Negative | Negative | 0.09 | Negative |  |  |  |
| 622 | 21-Oct-20 | Negative | Negative | 0.09 | Negative |  |  |  |
| 623 | 19-Oct-20 | Negative | Negative | 0.09 | Negative |  |  |  |
| 624 | 20-Oct-20 | Negative | Negative | 0.09 | Negative |  |  |  |
| 625 | 21-Oct-20 | Negative | Negative | 0.09 | Negative |  |  |  |
| 626 | 19-Oct-20 | Negative | Negative | 0.09 | Negative |  |  |  |
| 627 | 19-Oct-20 | Negative | Negative | 0.09 | Negative |  |  |  |
| 628 | 21-Oct-20 | Negative | Negative | 0.09 | Negative |  |  |  |
| 629 | 21-Oct-20 | Negative | Negative | 0.09 | Negative |  |  |  |
| 630 | 19-Oct-20 | Negative | Negative | 0.09 | Negative |  |  |  |
| 631 | 21-Oct-20 | Negative | Negative | 0.09 | Negative |  |  |  |
| 632 | 19-Oct-20 | Negative | Negative | 0.09 | Negative |  |  |  |
| 633 | 19-Oct-20 | Negative | Negative | 0.09 | Negative |  |  |  |
| 634 | 19-Oct-20 | Negative | Negative | 0.09 | Negative |  |  |  |
| 635 | 19-Oct-20 | Negative | Negative | 0.09 | Negative |  |  |  |
| 636 | 19-Oct-20 | Negative | Negative | 0.09 | Negative |  |  |  |
| 637 | 19-Oct-20 | Negative | Negative | 0.09 | Negative |  |  |  |
| 638 | 21-Oct-20 | Negative | Negative | 0.09 | Negative |  |  |  |
| 639 | 21-Oct-20 | Negative | Negative | 0.09 | Negative |  |  |  |
| 640 | 20-Oct-20 | Negative | Negative | 0.09 | Negative |  |  |  |
| 641 | 20-Oct-20 | Negative | Negative | 0.09 | Negative |  |  |  |
| 642 | 21-Oct-20 | Negative | Negative | 0.09 | Negative |  |  |  |
| 643 | 21-Oct-20 | Negative | Negative | 0.09 | Negative |  |  |  |
| 644 | 21-Oct-20 | Negative | Negative | 0.09 | Negative |  |  |  |
| 645 | 19-Oct-20 | Negative | Negative | 0.09 | Negative |  |  |  |
| 646 | 19-Oct-20 | Negative | Negative | 0.09 | Negative |  |  |  |
| 647 | 19-Oct-20 | Negative | Negative | 0.09 | Negative |  |  |  |
| 648 | 21-Oct-20 | Negative | Negative | 0.09 | Negative |  |  |  |
| 649 | 21-Oct-20 | Negative | Negative | 0.09 | Negative |  |  |  |
| 650 | 20-Oct-20 | Negative | Negative | 0.09 | Negative |  |  |  |
| 651 | 20-Oct-20 | Negative | Negative | 0.09 | Negative |  |  |  |
| 652 | 19-Oct-20 | Negative | Negative | 0.09 | Negative |  |  |  |
| 653 | 21-Oct-20 | Negative | Negative | 0.09 | Negative |  |  |  |
| 654 | 21-Oct-20 | Negative | Negative | 0.09 | Negative |  |  |  |
| 655 | 20-Oct-20 | Negative | Negative | 0.09 | Negative |  |  |  |
| 656 | 20-Oct-20 | Negative | Negative | 0.09 | Negative |  |  |  |
| 657 | 20-Oct-20 | Negative | Negative | 0.09 | Negative |  |  |  |
| 658 | 19-Oct-20 | Negative | Negative | 0.09 | Negative |  |  |  |
| 659 | 20-Oct-20 | Negative | Negative | 0.09 | Negative |  |  |  |
| 660 | 21-Oct-20 | Negative | Negative | 0.09 | Negative |  |  |  |
| 661 | 21-Oct-20 | Negative | Negative | 0.09 | Negative |  |  |  |
| 662 | 21-Oct-20 | Negative | Negative | 0.09 | Negative |  |  |  |
| 663 | 21-Oct-20 | Negative | Negative | 0.09 | Negative |  |  |  |
| 664 | 21-Oct-20 | Negative | Negative | 0.09 | Negative |  |  |  |
| 665 | 20-Oct-20 | Negative | Negative | 0.09 | Negative |  |  |  |
| 666 | 20-Oct-20 | Negative | Negative | 0.09 | Negative |  |  |  |
| 667 | 19-Oct-20 | Negative | Negative | 0.09 | Negative |  |  |  |
| 668 | 19-Oct-20 | Negative | Negative | 0.09 | Negative |  |  |  |
| 669 | 20-Oct-20 | Negative | Negative | 0.09 | Negative |  |  |  |
| 670 | 20-Oct-20 | Negative | Negative | 0.09 | Negative |  |  |  |
| 671 | 20-Oct-20 | Negative | Negative | 0.09 | Negative |  |  |  |
| 672 | 20-Oct-20 | Negative | Negative | 0.09 | Negative | 4-Nov-20 | 19 |  |
| 673 | 19-Oct-20 | Negative | Negative | 0.09 | Negative | 19-Oct-20 | 19.69 |  |
| 674 | 19-Oct-20 | Negative | Negative | 0.09 | Negative | 21-May-20 | 35.73 |  |
| 675 | 19-Oct-20 | Negative | Negative | 0.08 | Negative |  |  |  |
| 676 | 21-Oct-20 | Negative | Negative | 0.08 | Negative |  |  |  |
| 677 | 21-Oct-20 | Negative | Negative | 0.08 | Negative |  |  |  |
| 678 | 21-Oct-20 | Negative | Negative | 0.08 | Negative |  |  |  |
| 679 | 21-Oct-20 | Negative | Negative | 0.08 | Negative |  |  |  |
| 680 | 21-Oct-20 | Negative | Negative | 0.08 | Negative |  |  |  |
| 681 | 21-Oct-20 | Negative | Negative | 0.08 | Negative |  |  |  |
| 682 | 19-Oct-20 | Negative | Negative | 0.08 | Negative |  |  |  |
| 683 | 21-Oct-20 | Negative | Negative | 0.08 | Negative |  |  |  |
| 684 | 19-Oct-20 | Negative | Negative | 0.08 | Negative |  |  |  |
| 685 | 20-Oct-20 | Negative | Negative | 0.08 | Negative |  |  |  |
| 686 | 21-Oct-20 | Negative | Negative | 0.08 | Negative |  |  |  |
| 687 | 21-Oct-20 | Negative | Negative | 0.08 | Negative |  |  |  |
| 688 | 21-Oct-20 | Negative | Negative | 0.08 | Negative |  |  |  |
| 689 | 19-Oct-20 | Negative | Negative | 0.08 | Negative |  |  |  |
| 690 | 21-Oct-20 | Negative | Negative | 0.08 | Negative |  |  |  |
| 691 | 19-Oct-20 | Negative | Negative | 0.08 | Negative |  |  |  |
| 692 | 21-Oct-20 | Negative | Negative | 0.08 | Negative |  |  |  |
| 693 | 21-Oct-20 | Negative | Negative | 0.08 | Negative |  |  |  |
| 694 | 21-Oct-20 | Negative | Negative | 0.08 | Negative |  |  |  |
| 695 | 19-Oct-20 | Negative | Negative | 0.08 | Negative |  |  |  |
| 696 | 19-Oct-20 | Negative | Negative | 0.08 | Negative |  |  |  |
| 697 | 21-Oct-20 | Negative | Negative | 0.08 | Negative |  |  |  |
| 698 | 20-Oct-20 | Negative | Negative | 0.08 | Negative |  |  |  |
| 699 | 21-Oct-20 | Negative | Negative | 0.08 | Negative |  |  |  |
| 700 | 20-Oct-20 | Negative | Negative | 0.08 | Negative |  |  |  |
| 701 | 21-Oct-20 | Negative | Negative | 0.08 | Negative |  |  |  |
| 702 | 20-Oct-20 | Negative | Negative | 0.08 | Negative |  |  |  |
| 703 | 21-Oct-20 | Negative | Negative | 0.08 | Negative |  |  |  |
| 704 | 19-Oct-20 | Negative | Negative | 0.08 | Negative |  |  |  |
| 705 | 20-Oct-20 | Negative | Negative | 0.08 | Negative |  |  |  |
| 706 | 20-Oct-20 | Negative | Negative | 0.08 | Negative |  |  |  |
| 707 | 20-Oct-20 | Negative | Negative | 0.08 | Negative |  |  |  |
| 708 | 20-Oct-20 | Negative | Negative | 0.08 | Negative |  |  |  |
| 709 | 21-Oct-20 | Negative | Negative | 0.08 | Negative |  |  |  |

Ct-cycle threshold; N/R-not reported; N/A-not applicable; PCR-polymerase chain reaction.

*BioMedomics COVID-19 IgM/IgG Rapid Test assay results were generated by reading the marked red detection line(s) [1].

**Roche Elecsys Anti SARS-CoV-2 assay results were generated using reactive for optical density cutoff index ≥1.0 vs. non-reactive for cutoff index <1.0 [2].

^#^Specimen number ordered by descending optical density value (antibody titers) of the Roche Elecsys Anti SARS-CoV-2 assay.

^$^Severity per WHO classification [3]. If N/A, no severity classification was conducted due to absence of serious symptoms to require hospitalization and severity assessment.

**References**

1. BioMedomics I. COVID-19 IgM/IgG Rapid Test. 2020. [November 1, 2020]. Available from: <https://www.biomedomics.com/products/infectious-disease/covid-19-rt/>.

2. The Roche Group. Roche’s COVID-19 antibody test receives FDA Emergency Use Authorization and is available in markets accepting the CE mark. 2020. [June 5, 2020]. Available from: <https://www.roche.com/media/releases/med-cor-2020-05-03.htm>.

3. World Health Organization. Clinical management of COVID-19. Available from: <https://www.who.int/publications-detail/clinical-management-of-covid-19>. Accessed on: May 31st 2020. 2020.
