# Supplementary material for: Assessing the performance of a serological point-of-care test in measuring detectable antibodies against SARS-CoV-2
Source: PLoS One. 2022 Jan 31;17(1):e0262897. doi: 10.1371/journal.pone.0262897 (PMC8803198; doi:10.1371/journal.pone.0262897)
Supplement: S2 Table — (DOCX) [file pone.0262897.s002.docx]

**Assessing the performance of a serological point-of-care test in measuring detectable antibodies against SARS-CoV-2**

Supporting Information

**Table S2.** Concordance metrics between two SARS-CoV-2 antibody assays: the BioMedomics COVID-19 IgM/IgG Rapid Test and the Roche Elecsys Anti-SARS-CoV-2 including only specimens taken ≥14 days after the prior RT-PCR-confirmed infection.

| A) |  | **Roche Elecsys Anti-SARS-CoV-2** | | | **Overall percent agreement** | **Positive percent agreement** | **Negative percent agreement** | **Cohen's kappa statistic** |
| --- | --- | --- | --- | --- | --- | --- | --- | --- |
|  |  | **Positive** | **Negative** | **Total** | **% (95% CI)** | **% (95% CI)** | **% (95% CI)** | **k (95% CI)** |
| **BioMedomics COVID-19 IgM/IgG Rapid Test** | **Positive** | 432 | 0 | 432 | 91.8%  (89.5%-93.8%) | 88.5%  (85.3%-91.2%) | 100%  (98.1%-100%) | 0.82  (0.78-0.85) |
|  | **Negative** | 56 | 196 | 252 |  |  |  |  |
|  | **Total** | 488 | 196 | 684 |  |  |  |  |
