# Supplementary material for: Assessing the performance of a serological point-of-care test in measuring detectable antibodies against SARS-CoV-2
Source: PLoS One. 2022 Jan 31;17(1):e0262897. doi: 10.1371/journal.pone.0262897 (PMC8803198; doi:10.1371/journal.pone.0262897)
Supplement: S3 Table — (DOCX) [file pone.0262897.s003.docx]

**Assessing the performance of a serological point-of-care test in measuring detectable antibodies against SARS-CoV-2**

Supporting Information

**Table S3.** Results of univariable and multivariable logistic regression, assessing the association between seropositivity using the BioMedomics COVID-19 IgM/IgG Rapid Test and the following covariates: RT-PCR Ct value (cut-off at 35), Elecsys optical density value, and severity of infection.

|  |  | **Crude OR (95% CI)** | **P-value** | **aOR (95% CI)** | **P-value** |
| --- | --- | --- | --- | --- | --- |
| **PCR cycle threshold (Ct) value** | **Low Ct value (<35)** | Ref |  | Ref |  |
|  | **High Ct value (≥35)** | 0.11 (0.03-0.45) | 0.002 | 0.15 (0.03-0.78) | 0.024 |
|  | **No PCR/No Ct value** | 0.08 (0.05-0.14) | <0.001 | 0.35 (0.18-0.67) | 0.001 |
| **Optical density value of the Roche Elecsys Anti SARS-CoV-2 assay** | **Lower antibody titers (optical density value <10.0)** | Ref |  | Ref |  |
|  | **Higher antibody titers (≥10.0)** | 5.03 (2.80-9.02) | <0.001 | 5.60 (3.04-10.34) | <0.001 |
|  | **Negative result (no optical density value)** | N/A | N/A | N/A | N/A |
| **Severity^$^** | **Non-severe (asymptomatic, mild, or moderate infection)** | Ref |  | Ref |  |
|  | **Severe (severe or critical infection)** | 5.68 (1.31-24.58) | <0.020 | 0.94 (0.19-4.60) | 0.938 |

**References**

1. BioMedomics I. COVID-19 IgM/IgG Rapid Test. 2020. [November 1, 2020]. Available from: <https://www.biomedomics.com/products/infectious-disease/covid-19-rt/>.

2. The Roche Group. Roche’s COVID-19 antibody test receives FDA Emergency Use Authorization and is available in markets accepting the CE mark. 2020. [June 5, 2020]. Available from: <https://www.roche.com/media/releases/med-cor-2020-05-03.htm>.

3. World Health Organization. Clinical management of COVID-19. Available from: <https://www.who.int/publications-detail/clinical-management-of-covid-19>. Accessed on: May 31st 2020. 2020.
